# Collaborative rewiring of the pluripotency network by chromatin and signalling modulating pathways

**DOI:** 10.1038/ncomms7188

**Published:** 2015-02-04

**Authors:** Khoa A. Tran, Steven A. Jackson, Zachariah P.G. Olufs, Nur Zafirah Zaidan, Ning Leng, Christina Kendziorski, Sushmita Roy, Rupa Sridharan

**Affiliations:** 1 Wisconsin Institute for Discovery, University of Wisconsin, Madison, 330 N. Orchard Street, Room 2118, Wisconsin 53715, USA; 2 Molecular and Cellular Pharmacology Program, University of Wisconsin, Madison, 330 N. Orchard Street, Room 2118, Wisconsin 53715, USA; 3 Department of Statistics, University of Wisconsin, Madison, 330 N. Orchard Street, Room 2118, Wisconsin 53715, USA; 4 Department of Biostatistics and Medical Informatics, University of Wisconsin, Madison, 330 N. Orchard Street, Room 2118, Wisconsin 53715, USA; 5 Department of Cell and Regenerative Biology, University of Wisconsin, Madison, 330 N. Orchard Street, Room 2118, Wisconsin 53715, USA

## Abstract

Reprogramming of somatic cells to induced pluripotent stem cells (iPSCs) represents a profound change in cell fate. Here, we show that combining ascorbic acid (AA) and 2i (MAP kinase and GSK inhibitors) increases the efficiency of reprogramming from fibroblasts and synergistically enhances conversion of partially reprogrammed intermediates to the iPSC state. AA and 2i induce differential transcriptional responses, each leading to the activation of specific pluripotency loci. A unique cohort of pluripotency genes including *Esrrb* require both stimuli for activation. Temporally, AA-dependent histone demethylase effects are important early, whereas Tet enzyme effects are required throughout the conversion. 2i function could partially be replaced by depletion of components of the epidermal growth factor (EGF) and insulin growth factor pathways, indicating that they act as barriers to reprogramming. Accordingly, reduction in the levels of the EGF receptor gene contributes to the activation of *Esrrb*. These results provide insight into the rewiring of the pluripotency network at the late stage of reprogramming.

## Abstract (web summary)

Improving the efficiency of reprogramming of somatic cells to induced pluripotent stem cells is of major interest. Here, the authors combine ascorbic acid and 2i (MAP kinase and GSK inhibitors) conditions and show increased efficiency and synchronicity in the reprogramming of fibroblasts and partially reprogrammed cells, and study epigenetic effectors and signalling pathways responsible for this effect.

## Graphical abstract

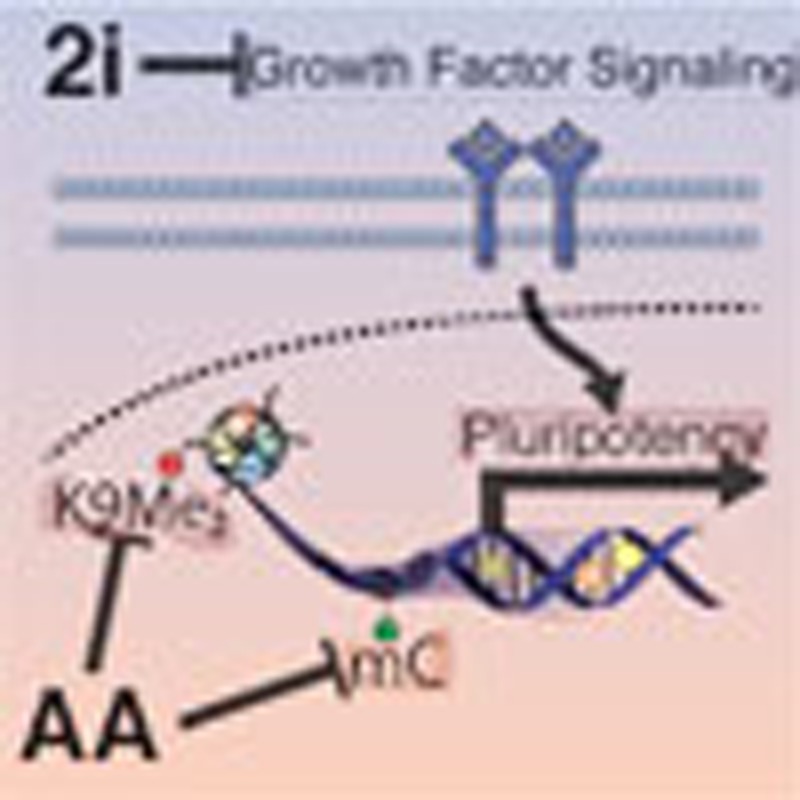

Transcription factor-mediated reprogramming converts somatic cells to induced pluripotent stem cells (iPSCs)^1^. This process takes several days, during which both the epigenome and the transcriptome of somatic cells are reset to a pluripotent state^2^,^3^. Recent progress has demonstrated that there are two waves of transcriptional change, one immediately following the induction of the reprogramming factors and the other occurring later to activate the pluripotency gene network^4^. At the level of the epigenome, although some locus-specific chromatin marks are established early^5^, global changes in histone modification profiles^6^ and resetting of DNA methylation^4^ occur at later stages in the reprogramming process, concomitant with activation of pluripotency network. How this final step is established remains unknown and is hampered both by the heterogeneity in the reprogramming populations^7^ and multiple routes that can be used to reach this state^8^.

The efficiency and fidelity of the reprogramming process can be improved by components present in the media. Such components belong to two main classes: epigenetic modifiers such as valproic acid (VPA)^9^, ascorbic acid (AA)^10^,^11^,^12^,^13^,^14^ and 5-azacytidine^15^,^16^,^17^ and modulators of signalling such as transforming growth factor (TGF)-β inhibitors (TGF-βi)^13^,^18^ and components of the 2i media^13^,^14^,^16^,^19^. 2i is the minimal medium sufficient to propagate mouse embryonic stem cells (ESCs) and is the combination of a mitogen-activated protein kinase inhibitor which can repress differentiation signals and a glycogen synthetase kinase inhibitor (GSKi) that improves clonogenecity^20^. Recent reports suggest that combination of AA with GSKi^14^ and/or TGF-βi^13^ greatly improved reprogramming efficiency especially when starting from blood progenitor cells.

We found that combining an epigenetic modifier (AA) and signalling modifier (2i) significantly enhances the efficiency of reprogramming from embryonic and adult somatic cells and converted clonal partially reprogrammed (pre-iPSC) lines to iPSCs at up to 80% levels. AA can function as a cofactor of several epigenetic enzymes that contain an Fe-S core and include both histone demethylases and the Tet enzymes that hydroxymethylate 5-methyl cytosine (5mC) on DNA providing a pathway for active DNA demethylation. Besides increasing reprogramming efficiency, AA was also found to improve the quality of mouse embryonic fibroblast (MEF)-derived iPSCs. iPSCs obtained with a particular reprogramming factor stoichiometry^21^,^22^ displayed aberrant silencing of the imprinted *Dlk-Dio* cluster^21^, leading to poor contribution to chimeras in a tetraploid complementation assay, which was relieved by culture in AA-containing media. Similarly, ESCs propagated in 2i have a more hypomethylated genome that resembles more faithfully the pre-implantation epiblast^23^,^24^,^25^,^26^,^27^.

Using this high efficiency conversion system, we specifically focused on delineating the mechanism of rewiring of the pluripotency network at the end of reprogramming. By performing genome-wide transcriptional analysis, we found that AA mainly activated, whereas 2i contributed to downregulation of genes that were important for the transition to the iPSC state. If AA and 2i were added in a non-overlapping manner, AA had to precede 2i addition. Temporally, histone demethylase activity was required early during the conversion. By contrast, Tet enzyme levels that mediate DNA hydroxymethylation had to be maintained throughout the conversion to the iPSC state. Some components of the transcriptional circuitry responded to the AA stimulus alone—*Nanog, Zfp42, Utf1*—or signalling alone—*Tcfcp2l1*, endogenous *Klf4*—whereas both activities were required for activation of *Esrrb* and *Tbx3*. Network analysis identified epidermal growth factor and insulin-like growth factor signalling as key targets of 2i for downregulation. Functionally depleting members of these pathways could partially replace 2i, suggesting that they are barriers to reprogramming. Strikingly, reducing levels of *Egfr* contributed to the upregulation of *Esrrb*. Taken together, our results reveal the requirements for the activation of the pluripotency network in the late stages of reprogramming.

## Results

### AA+2i leads to highly efficient conversion to iPSCs

**Figure 1:**
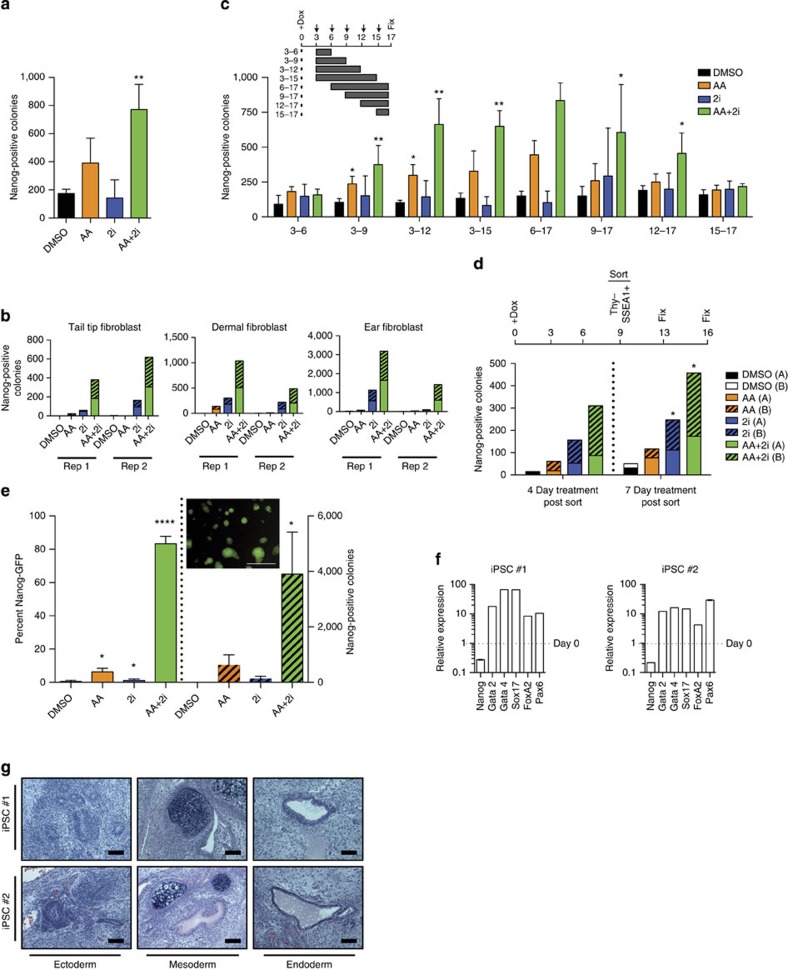
AA combined with 2i enhances reprogramming efficiency. (**a**) Counts of Nanog-positive colonies on day 17 of MEF reprogramming. Error bars represent standard deviation of four replicates. AA, ascorbic acid; 2i, MAP kinase inhibitor (PD-0325901)+glycogen synthetase kinase inhibitor (CHIR-99021). (**b**) Same as above but for adult fibroblasts. Technical replicates are stacked. Rep1 and Rep2 indicate two different genetic backgrounds. (**c**) Inset—Scheme of experiment: JSS MEFs received doxycycline (dox) for OSKM induction from day 0 to day 17 and were treated with DMSO, AA, 2i or AA+2i for the days indicated (grey bars). Counts of Nanog-positive colonies for four replicates. *P<0.05 **P<0.01 assessed by *t*-test. (**d**) Top panel—Scheme of experiment: Cells were sorted for SSEA1 and lack of Thy1 expression (Thy1^−^SSEA1^+^) on day 9 after dox induction. Sorted cells were exposed to DMSO, AA, 2i or AA+2i until day 13 or 16 post dox induction. Bottom panel—Bar graphs show combined results from two biological replicates (A and B) for Nanog-positive colonies after 4 (left) or 7 (right) days of treatment. *P<0.05 by *t*-test. (**e**) Left panel—Bar graph representing GFP reporter expression (under the control of the endogenous Nanog promoter) in pre-iPSCs upon exposure to AA and/or 2i as compared with DMSO by flow cytometry. Right panel—Counts of Nanog-GFP-positive ESC-like colonies that represent the Nanog-GFP-positive cells in the left panel. Inset: Image of Nanog-GFP-positive colonies upon exposure to AA+2i. Scale bar, 250 μm. Error bars represent standard deviation of three biological replicates. Asterisk indicates significance *P<0.05, ****P<0.0001 assessed by *t*-test. (**f**) Quantitative reverse transcription—PCR of various differentiation markers on day 7 post *in vitro* differentiation of two independent iPSC lines #1 and #2 derived from pre-iPSCs after AA+2i treatment. Dotted line represents levels on day 0 which was set to 1. (**g**) Images depicting the three germ layers from teratomas formed by two independent AA+2i-derived iPSC clones. Scale bar, 100 μm.

We applied a combination of the chromatin modifier (AA) and a signalling inhibitor (2i) to the reprogramming of MEFs that were homozygous for the reprogramming allele (four Yamanaka factors Oct4, Sox2, c-Myc and Klf4 integrated at a single locus in the genome) under the control of a doxycycline (dox)-inducible promoter and heterozygous for the tetracycline reverse transcriptional activator (rtta)^28^,^29^. Reprogramming factors were induced for 3 days in serum/leukaemia inhibitory factor (LIF)-containing ESC media after which the media were supplemented with control dimethylsulphoxide (DMSO), AA, 2i or AA+2i. The AA+2i condition lead to significantly increased reprogramming efficiency assessed by Nanog immunostaining (Fig. 1a). A similar effect was observed with adult tail-tip, dermal and ear fibroblasts with AA+2i yielding greatly more Nanog-positive iPSC colonies than either AA or 2i alone, although the response to individual stimuli were different from MEFs (Fig. 1b). Longer exposure of the reprogramming MEFs to AA+2i increased the number of reprogrammed colonies (Fig. 1c). As the highest number of colonies always included the window of treatment between day 9 and 12, we tested whether reprogramming intermediates in this interval were also responsive to these treatments.

Intermediate populations that had lost somatic expression, identified by lack of fibroblast marker Thy1, and gained intermediate markers of pluripotency, defined by SSEA1 expression, were isolated on day 9 of reprogramming by flow cytometry (Supplementary Fig. 1a). There was an increase in the number of colonies obtained from Thy1^−^SSEA^+^ cells in the AA+2i combination to almost 20-fold over control conditions (Fig. 1d). As the Thy1^−^SSEA^+^ cells represent a heterogeneous mixture of reprogramming cells, we tested whether we could observe similar effects with reprogramming intermediates from previously derived homogenous clonal pre-iPSC lines.

Pre-iPSCs are stalled reprogramming intermediates that have an ESC-like morphology^19^,^30^. They display both a gene expression pattern^30^ and three-dimensional chromatin conformation^31^ intermediate to that of a somatic cell and an iPSC; and have not yet changed their global epigenome as defined by histone modifications to a pluripotent profile^6^. These clonal pre-iPSC lines had undergone the first wave of transcriptional changes, express E-cadherin (Supplementary Fig. 1b) and SSEA1 on their cell surface^30^. Global expression profile of these pre-iPSCs closely clustered with day 9 and 12 Thy1^−^SSEA^+^ cells (Supplementary Fig. 1c)^4^, confirming the notion that they resembled the late stage of reprogramming. Remarkably, the combination of AA and 2i (Supplementary Fig. 1b) led to a tremendous synergistic conversion of pre-iPSCs to up to 80% Nanog-green fluorescent protein (GFP)-positive cells representing ESC-like colonies in 10 days (Fig. 1e). This was the case for pre-iPSCs derived from both MEFs and astrocytes (Supplementary Fig. 1d,e). There was minimal conversion to the iPSC state in the presence of AA (~5%) or 2i alone (2%; Fig. 1e and Supplementary Fig. 1d). GFP-positive colonies could be passaged for greater than five generations in the absence of AA+2i (Supplementary Fig. 1f). Clonal iPSC lines derived in the AA+2i conditions had activated endogenous expression of *Oct4, Sox2, Esrrb* and Pecam1 (Supplementary Fig. 1g,i) and extinguished exogenous reprogramming factor expression (Supplementary Fig. 1h). Importantly, these clonal lines could be *in vitro* differentiated into all three germ layers (Fig. 1f) and when injected *in vivo* gave rise to teratomas that represented all three germ layers (Fig. 1g).

### AA activity is required to precede 2i exposure

**Figure 2:**
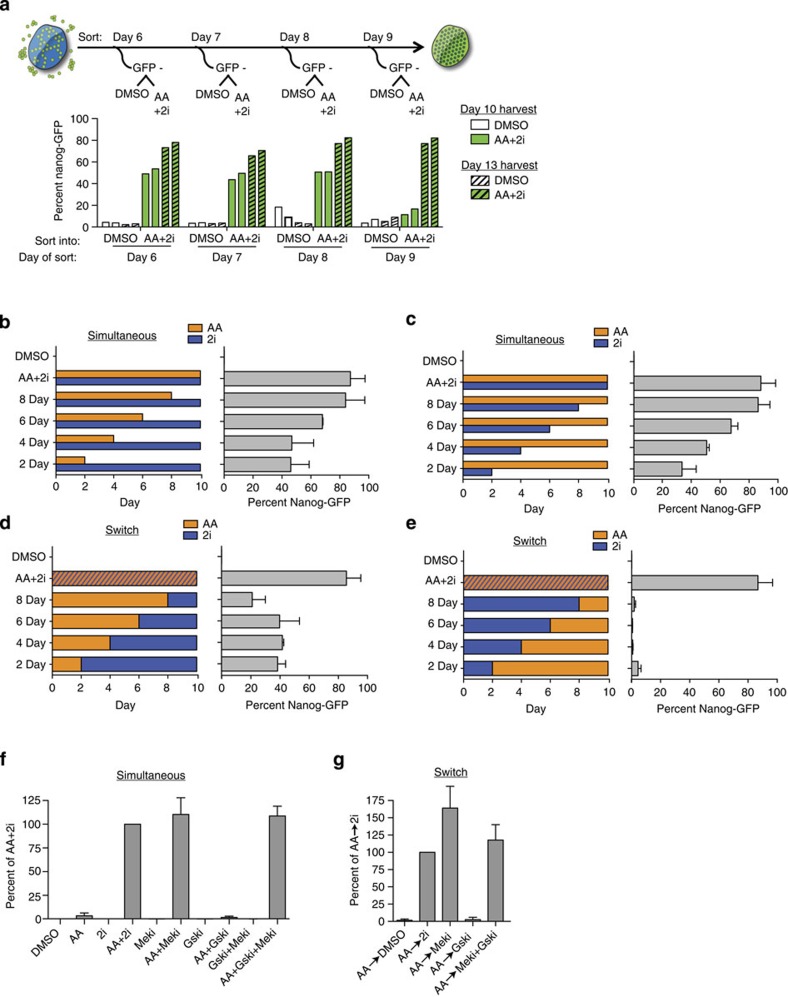
Different temporal requirements for AA and 2i. (**a**) Top panel: Scheme of experiment: GFP-negative cells (GFP-) were sorted by flow cytometry on the indicated days into wells containing DMSO or AA+2i and analysed for Nanog-GFP-positive cells on day 10 and day 13. Bottom panel: Nanog-GFP-positive cells derived from Nanog-GFP-negative cells sorted on days indicated on the *x* axis, cultured in DMSO or AA+2i, and analysed on day 10 (solid bars) or day 13 (hatched bars). Individual bars represent independent replicates. (**b**) Scheme of experiment (left panel) indicating the number of days of AA exposure when 2i was retained for all 10 days and corresponding Nanog-GFP-positive conversion rates obtained (right panel). Error bars represent standard deviation of four replicates. (**c**) Scheme of experiment (left panel) indicating the number of days of 2i exposure when AA was retained for all 10 days and corresponding Nanog-GFP-positive conversion rates obtained on day 10 (right panel). Error bars represent standard deviation of four replicates. (**d**) Scheme of experiment (left panel) indicating the number of days of AA exposure alone followed by switch to medium containing 2i alone and corresponding Nanog-GFP-positive conversion rates obtained (right panel). Error bars represent standard deviation of four replicates. (**e**) Scheme of experiment (left panel) indicating the number of days of 2i exposure alone followed by switch to medium containing AA alone and corresponding Nanog-GFP-positive conversion rates obtained (right panel). Error bars represent standard deviation of four replicates. (**f**) Nanog-GFP-positive conversion rates obtained when pre-iPSCs were exposed to AA and individual components of 2i—Meki and Gski. Error bars represent standard deviation of three biological replicates. (**g**) Nanog-GFP-positive conversion rates obtained when pre-iPSCs were exposed AA for initial 2 days and then switched to individual or combined components of 2i for 8 days. Error bars represent standard deviation of three biological replicates.

The number of Nanog-GFP-positive cells increased gradually during reprogramming from day 4 onwards (Supplementary Fig. 2a,b), with early emerging colonies (day 6) expressing Esrrb, suggesting complete reprogramming (Supplementary Fig. 2b). We sorted the Nanog-GFP-negative populations from day 6 onwards into either a control DMSO or the AA+2i condition (Fig. 2a). By day 10, 50% of the GFP-negative population had converted to a GFP-positive state, which extended to 80% on day 13 (Fig. 2a). Under any treatment, the cells grew slower than in the DMSO condition, but there was no significant cell death compared with DMSO (Supplementary Fig. 2c,d). These observations suggest that almost the entire population of pre-iPSCs transitioned to the iPSC state.

To start gaining insight into the mechanism of the conversion, we exposed pre-iPSCs to both AA and 2i at the start of the experiment, with one component either AA or 2i removed at 2-day intervals up to 10 days (Fig. 2b,c). There was a gradual increase in the number of iPSCs obtained proportional to the number of days that the cells were exposed to both components, irrespective of whether AA or 2i was removed (Fig. 2b,c), suggesting that there was a continued requirement for both factors to achieve maximal conversion. We then applied AA or 2i in a non-overlapping manner (Fig. 2d,e). About half of maximal conversion was attained when cells were first exposed to AA for just 2 days followed by a switch to media containing 2i (Fig. 2d). Increased exposure to AA alone beyond 2 days did not improve reprogramming efficiency. Conversion rates reduced if AA was applied for the initial 8 days and then switched to media containing to 2i for 2 days (Fig. 2d), but improved with increasing length of 2i exposure (Supplementary Fig. 2e). In stark contrast to these results, if 2i exposure preceded AA exposure, less than 2% of the cells converted at the end of 10 days (Fig. 2e). This suggests that exposure to AA was either required for the action of 2i-mediated effects or pre-treatment with 2i-inhibited AA effects.

To determine which of the inhibitors in 2i was important for pre-iPSC to iPSC conversion, we added either the MEK inhibitor or the GSK inhibitor in the presence of AA. In both the simultaneous (Fig. 2f, Supplementary Fig. 2f) and switch conditions (Fig. 2g, Supplementary Fig. 2g), the MEK inhibitor was essential for the conversion, although the GSK inhibitor improved both the appearance and the number of compact colonies (Supplementary Fig. 2h). Therefore in subsequent experiments, we continued to use the AA+2i combination.

### Requirement for H3K9 demethylase and Tet activities differs

AA can act as a cofactor for several chromatin-modifying enzymes that contain an Fe-S core^32^, including the jumonji-domain-containing histone demethylases and the Tet enzymes, which convert 5mC to 5-hydroxymethylcytosine (5hmC) providing a pathway for active DNA demethylation. Of the enzymes thought to be involved in AA function during reprogramming^10^,^11^, we concentrated on those involved in H3K9 methylation erasure^6^,^10^, as this occurs at the late stage of reprogramming^6^. The Tet enzymes have roles to play in reprogramming^33^, both in the mesenchymal-to-epithelial transition^34^ and in iPSC transition^35^ and can even replace Oct4 function^36^.

We first tested whether AA function could be replaced by epigenome-modifying activities in the context of 2i-mediated reprogramming. Subjecting the cells to 5-azacytidine a DNA methyltransferase inhibitor in the presence of 2i led to a 30% conversion efficiency (Supplementary Fig. 3b), albeit with higher cell death. Similarly, short interfering RNA (siRNA)-mediated depletion of the H3K9 methyltransferases *Ehmt1 (Glp)* or *Ehmt2 (G9a)* (Supplementary Fig. 3a,c) resulted in about 30–40% conversion in the presence of 2i. Thus, both the histone and DNA demethylating pathways contribute to AA-mediated effects in the presence of 2i.

**Figure 3:**
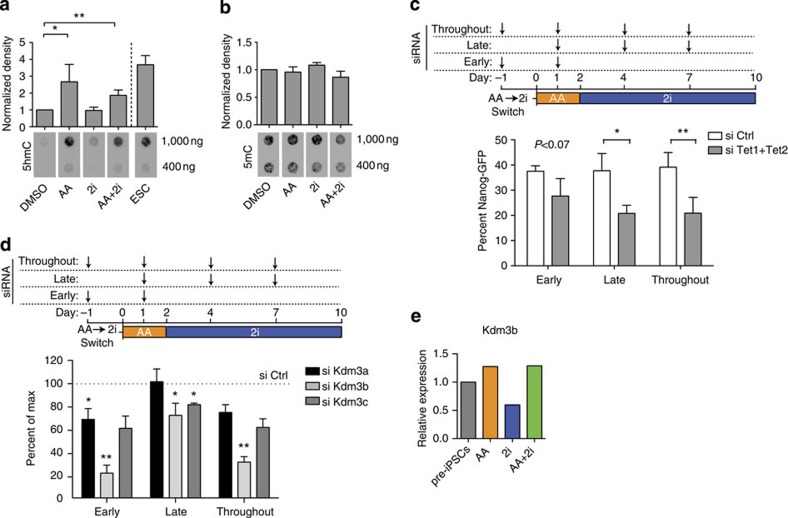
Histone demethylase effects of AA are essential early. (**a**) Quantification of 5hmC levels normalized to DMSO control (top panel) of the dot blot of 5hmC levels (bottom panel) under DMSO, AA, 2i or AA+2i conditions for 2 days, compared with untreated ESC level. Error bars represent standard deviation of four biological replicates. Asterisk indicates significance *P<0.05, **P<0.01 assessed by *t*-test. (**b**) Quantification of 5mC levels normalized to DMSO control (top panel) of the dot blot of 5mC levels (bottom panel) under DMSO, AA, 2i or AA+2i conditions for 2 days. (**c**) Top panel—Scheme of the experiment: siRNA transfections targeting both Tet1 and Tet2 or control (anti-luciferase) were performed early (days −1 and +1), late (days +1, +4 and +7) or throughout (days −1, +1, +4 and +7). Day of exposure to media containing AA alone was day 0. On day 2, media were switched to that containing 2i alone. Bottom panel—Quantification of Nanog-GFP-positive cells obtained on day 10 of the experiment. Error bars represent standard deviation from three biological replicates. Asterisk indicates significance *P<0.05, **P<0.01 assessed by *t*-test. (**d**) Top panel—Scheme of the experiment: siRNA transfections targeting histone demethylases or control (anti-luciferase) were performed early (days −1 and +1), late (days +1, +4 and +7) or throughout (days −1, +1, +4 and +7). Day of exposure to media containing AA alone was day 0. On day 2, media were switched to that containing 2i alone. Bottom panel—Quantification of Nanog-GFP-positive cells obtained on day 10 of the experiment upon knockdown of specific H3K9me1/me2 demethylases. Dotted line represents Nanog-GFP-positive levels obtained in control siRNA conditions set to 100%. Error bars represent standard deviation from three biological replicates. Asterisk indicates significance *P<0.05, **P<0.01 assessed by *t*-test. (**e**) Relative expression of Kdm3b in pre-iPSC treated with AA, 2i or AA+2i for 24 h.

We next assessed the global levels of 5hmC on day 2 upon exposure to AA, 2i or AA+2i as both AA and 2i have been shown to affect DNA hydroxymethylation levels in ESCs^24^,^25^,^26^,^27^,^37^ (Fig. 3a). Although exposure to AA caused a significant global increase in 5hmC on day 2 to levels seen in ESCs, exposure to 2i alone did not have an effect (Fig. 3a). These changes in 5hmC levels were not accompanied by significant changes in global 5mC levels (Fig. 3b).

To determine the requirement of the cognate enzymes, we depleted the Tet enzymes or the histone demethylases that act on H3K9me2/me1 during the AA+2i-mediated conversion. We found that the combined knockdown of both *Tet1* and *Tet2* had a stronger effect on reprogramming than knockdown of either transcript alone (Supplementary Fig. 3d). In contrast, there was a loss of reprogramming efficiency specifically upon reduction of histone demethylase *Kdm3b* levels (Supplementary Fig. 3e). We further corroborated the importance of *Kdm3b* by generating a Crispr-depleted *Kdm3b* cell line, and found that it could not be converted to the iPSC state in the presence of AA+2i (Supplementary Fig. 3f).

We then dissected the temporal requirements of these epigenetic activities using our switch system. By depleting *Tet1* and *Tet2* exclusively in the AA phase or the 2i phase, we asked whether there was a temporal difference in requirement for Tet transcript levels (Fig. 3c) There was a decrease in reprogramming efficiency following *Tet1+Tet2* knockdown in both the early and the late phases, suggesting that higher 5hmC levels (Fig. 3a) have to be sustained for iPSC conversion.

Performing similar experiments with the histone demethylases, there was an 80% decrease in reprogramming efficiency upon knockdown of Kdm3b in the early AA-dependent phase, but surprisingly a much more subtle effect on the late 2i-dependent phase (Fig. 3d). These results indicate that Kdm3b plays a key role in the early phase of the AA action. Interestingly, the expression of *Kdm3b* was reduced by twofold upon 24-h exposure to 2i (Fig. 3e), suggesting that this may contribute to the lack of reprogramming upon switching from 2i to AA (Fig. 2e).

These results suggest that of the two major epigenomic changes that have to occur in the late stage of reprogramming, the Kdm3b-mediated AA effects are important for the early phase, whereas the Tet-mediated effects need to be sustained in the 2i-dependent phase.

### AA and 2i drive divergent transcriptional output

**Figure 4:**
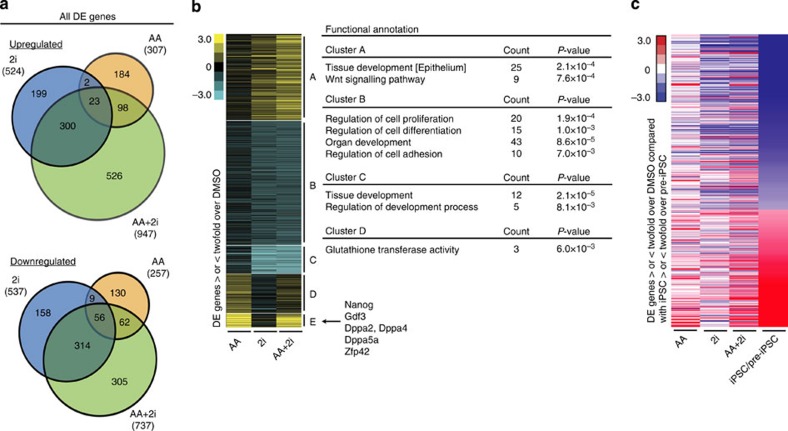
AA and 2i promote complementary transcriptional responses. (**a**) Venn diagrams showing the overlap of up- or downregulated differentially expressed (DE) transcripts between AA, 2i and AA+2i conditions on day 2 determined by EB-Seq. (**b**) K-means clustering for DE genes that had a twofold change over DMSO, grouped into five clusters, A–E. Gene ontology enrichment for each cluster is presented on the right. Stem cell-related genes within Cluster E are listed. Yellow=greater than DMSO, blue=lower than DMSO. (**c**) Heat-map showing relative expression of genes that are twofold differentially expressed between iPSCs and pre-iPSCs ordered from lowest to highest, compared with relative expression after 2 days of exposure to AA, 2i or AA+2i and DMSO. Red=higher in iPSCs or any treatment condition and blue=higher in pre-iPSCs or DMSO, respectively. Note: the iPSC to pre-iPSC ratio was calculated using raw values, whereas the AA, 2i and AA+2i conditions were DE genes that were twofold changed over DMSO.

We next wanted to gain insight into the molecular consequences that were triggered in response to the action of these agents. We chose day 2 as a suitable time point to perform genome-wide RNA sequencing as there were no Nanog-GFP colonies at this point providing us a snapshot of the transcriptional dynamics before conversion to the iPSC state occurred. Data from three biological replicates with high correlation (Supplementary Fig. 4a) were median normalized and used for identifying genes that are differentially expressed as compared with the control DMSO condition using the EB-seq method^38^. The advantage of this method is that it computes a posterior probability for differential expression (DE) and using a threshold of 0.95, a high confidence list of modulated genes was generated (Supplementary Data 1). Transcriptional changes in both directions were observed upon exposure to AA alone (307 up versus 257 down) and 2i alone (524 up versus 537 down; Fig. 4a). There was very little overlap between the genes that are modulated by AA and 2i (Fig. 4a). This divergent transcriptional response (Supplementary Fig. 4b) provides an explanation as to why each component is individually ineffective in mediating the high rate of conversion to iPSCs. However, for the set of genes that are common to all three conditions, the presence of both AA and 2i plays a role in amplifying the magnitude of changed expression (Supplementary Fig. 4c). In the AA+2i condition, almost half (526 up and 305 down) the genes were unique to the AA+2i condition, implying a novel synergistic response (Fig. 4a).

We applied a further twofold cutoff for expression change within the DE genes to recover the genes with the most significant change (Fig. 4b). Gene ontology analysis revealed that regulators of cell differentiation were downregulated in response to 2i (Cluster B), whereas 2i responsive upregulated genes were enriched for Wnt signalling (Cluster A). By contrast in the presence of AA, glutathione transferase activity was enriched (Cluster D). Interestingly, the cluster of genes that was strongly upregulated in AA or AA+2i (Cluster E) was not enriched for any single functional category but contained several genes related to pluripotency including *Nanog, Dppa2* and *Zfp42*.

We then compared the transcriptional response to changes during the pre-iPSC to iPSC conversion using previously published data^30^. Among genes that were twofold changed between pre-iPSCs and iPSCs, we found that the AA+2i condition (Pearson correlation 0.49) resembled the iPSC state the most as compared with AA alone (0.4) or 2i alone (0.38; Fig. 4c). Interestingly, the upregulated genes were already in an iPSC-like pattern on day 2 in the AA conditions and the most downregulated genes shared by the 2i conditions, again suggesting complementary responses of these stimuli (Fig. 4c).

### AA and 2i synergize to reconstruct the pluripotency network

Given the global nature of the transcriptional changes required for achieving the pluripotent state, we hypothesized that major components of the regulatory network associated with the pre-iPSC state need to be reset. We focused on the network that describes connectivity patterns among genes that are twofold DE in the AA+2i condition. As we currently lack accurate reconstructions of the cellular network in the pre-iPSC state, we utilized the STRING network^39^, which integrates both physical and functional interactions and overlaid the DE genes to obtain a dynamic snapshot on day 2 of the pre-iPSC to iPSC conversion at a stringent confidence level of 0.5 (Supplementary Data 2).

**Figure 5:**
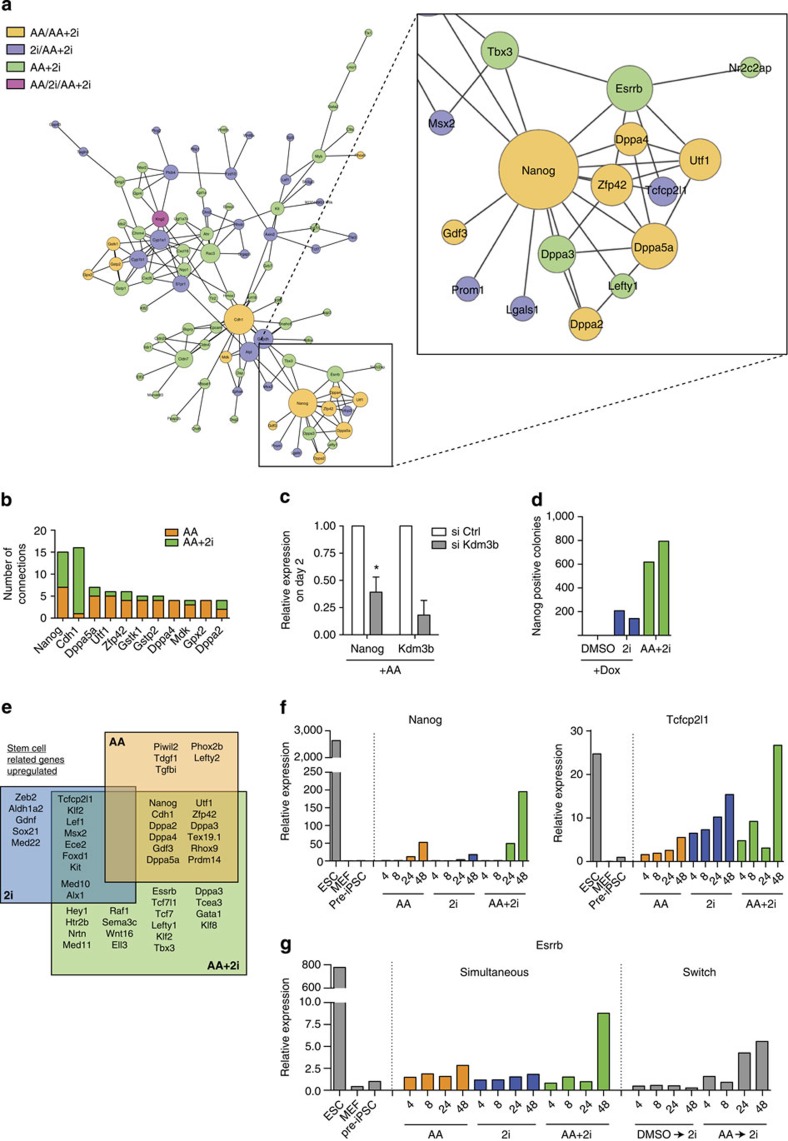
AA+2i increases connectivity of upregulated genes. (**a**) Networks generated from overlaying upregulated gene expression data onto STRING database. Nodes, representing genes, are identified by circles and their connections, or edges, by the lines joining them. Commonly regulated nodes cluster together, whereas loosely related nodes are at a greater distance. Size of the nodes is proportional to connectivity. A zoomed-in view of the Nanog subnetwork and its edges is presented. Orange=shared between the AA+2i and AA network, blue=shared between the AA+2i and 2i networks, green=unique to the AA+2i network. (**b**) Bar graph of upregulated nodes shared by AA (orange) and AA+2i (green). The number of edges for each node is indicated on the *y* axis. (**c**) Quantitative reverse transcription–PCR (qRT–PCR) data for expression of Nanog and Kdm3b following 2 days of exposure to AA combined with siRNA targeted against luciferase (si Ctrl) or Kdm3b. Error bars are standard deviation of three biological replicates. Asterisk indicates significance *P<0.05, by *t*-test. (**d**) Counts of Nanog-positive colonies obtained from Nanog-inducible pre-iPSC line in the presence of 2i alone as compared with control AA+2i condition. Data from two independent replicates are presented. (**e**) Venn diagrams of overlap between upregulated DE genes associated with maintenance of pluripotency. Genes in bold represent DE genes with greater than twofold expression change over DMSO. (**f**) qRT–PCR data over a 48-h time course of pluripotency genes responsive to AA or 2i alone following exposure to AA (orange), 2i (blue) or AA+2i (green). (**g**) qRT–PCR data for Esrrb expression analysed during a time-course exposure to AA (orange), 2i (blue) or AA+2i (green) or switch to 2i after either 48 h of DMSO or AA.

**Figure 6:**
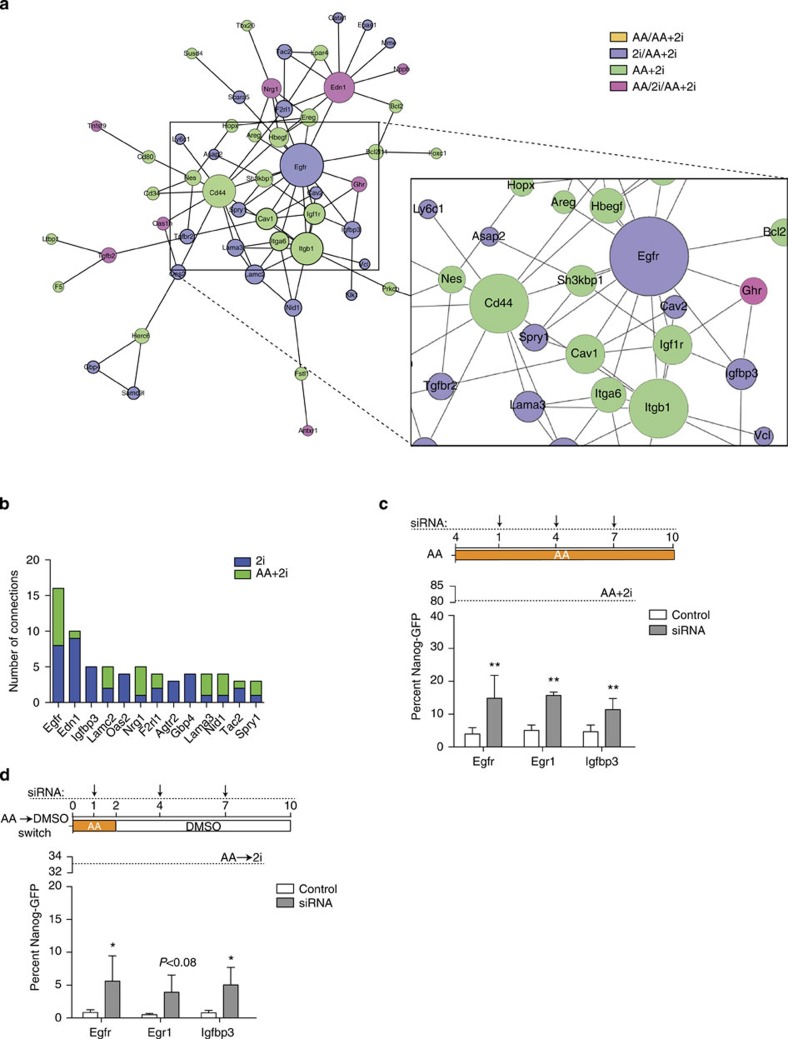
2i downregulated nodes are barriers to reprogramming. (**a**) Networks generated from overlaying downregulated gene expression data onto protein interaction from the STRING database. A zoomed-in view of the Egfr node and its edges is presented. Orange=shared between the AA+2i and AA network, blue=shared between the AA+2i and 2i networks, green=unique to the AA+2i network, pink=shared in all networks. (**b**) Bar graph of downregulated nodes shared by 2i (blue) and AA+2i (green). The number of edges for each node is indicated on the *y* axis. (**c**) Top panel—Scheme of the experiment: siRNA transfections targeting genes identified as key downregulated nodes or control (anti-luciferase) were performed on days +1, +4 and +7. Day of exposure to media containing AA alone was day 0. Bottom panel—Quantification of Nanog-GFP-positive cells obtained on day 10. Dotted line represents the per cent Nanog-GFP-positive cell levels obtained in control treatment (AA+2i). Error bars represent standard deviation from two to four biological replicates. **P<0.01 assessed by *t*-test. (**d**) Top panel—Scheme of the experiment: siRNA transfections targeting genes identified as key downregulated nodes or control (anti-luciferase) were performed on days +1, +4 and +7. Cells were treated with AA between day 0 and day 2 before switching to DMSO containing media until day 10. Bottom panel—Quantification of Nanog-GFP-positive cells obtained on day 10. Dotted line represents Nanog-GFP-positive levels obtained in control treatment (AA switched to 2i). Error bars represent standard deviation from three to five biological replicates. Asterisk indicates significance *P<0.05 assessed by *t*-test.

The AA+2i networks (Fig. 5a, Supplementary Fig. 5a, Fig. 6a and Supplementary Fig. 5b) were significantly enriched for interactions compared with networks generated from a random set of genes of the same size (up *z*-score=3.22541 and down *z*-score=2.68955), suggesting these genes are functionally related. Furthermore, genes that are commonly induced in AA+2i and AA are much more likely to connect to the genes that are uniquely induced in AA+2i (*z*-score of edges spanning these two gene sets=2.40702) than they are to genes that are uniquely induced in AA alone (*z*-score=1.86696; Fig. 5a and Supplementary Fig. 5c). This suggests that the common genes, which represent the core of the response to AA, are more functionally related to the remaining genes that form the AA+2i network. We observe a similar pattern of connectivity in the AA repressed and 2i-modulated sets (Fig. 6a and Supplementary Fig. 5d–f). This suggests that the genes induced or repressed uniquely in AA or 2i, and not in the AA+2i set, are less relevant to the larger transcriptional programme of cellular reprogramming.

In a network, genes (nodes) with a large number of connections, are associated with key regulatory functions. Strikingly, when we ranked the nodes in the AA+2i upregulated network, Nanog was the most highly connected and was already part of the AA alone network (Fig. 5b and Supplementary Data 2). Kdm3b was a key effector of the AA phase (Fig. 3d). We found that *Nanog* upregulation was severely diminished upon knockdown of *Kdm3b* in the presence of AA (Fig. 5c). Further, functionally, *Nanog* overexpression could partially replace AA function in 2i-mediated conversion of pre-iPSC to iPSC (Fig. 5d and Supplementary Fig. 5g), thus strengthening the predictions of our network analysis. Given this important role for Nanog, we also tested whether the members of the Nanog–proteomic interaction network were concomitantly upregulated in any condition but did not find a significant overlap (Supplementary Fig. 5h, i).

To examine how the network is reconstructed, we compared the upregulated genes to known regulators of stem cell maintenance. AA alone is able to increase expression of pluripotency genes such as *Nanog, Utf1* and *Zfp42* (Fig. 5e). However, upregulation of *Tcfcp2l1* and *Klf2* required 2i (Fig. 5e). Both AA and 2i are required for increased expression of *Esrrb, Tbx3* and *Tcf3* (Fig. 5e). Endogenous *Oct4* and *Sox2* were upregulated in AA conditions, whereas *Klf4* uniquely responded to 2i (Supplementary Fig. 5j). The expression of the reprogramming factors from the exogenous loci was repressed by day 2 in all conditions (Supplementary Fig. 5k). Thus, although from a global perspective (Fig. 4c) it appears that 2i is the driver of downregulating gene expression, there are important pluripotency genes that require 2i to upregulate expression.

Since several of these network components are reciprocally regulated, we next performed a time course to determine when representative genes of this multi-part rewiring were upregulated. An increase in AA-dependent *Nanog* expression was observed by 24 h (Fig. 5f). 2i-dependent *Tcfcp2l1* was upregulated within 4 h (Fig. 5f). Increased *Esrrb* expression was observed only 48 h after AA+2i treatment. Similar levels were reached when the cells were switched from AA media for 48 h to 2i exposure (Fig. 5g). This suggests that the two components can act sequentially to elevate levels of DE genes from the AA+2i condition.

### Downregulation of key nodes is functionally important

The most highly connected nodes in the AA+2i downregulated network, *Egfr*, *Igfbp3* and *Tgfβr2*, belonged to the growth-related signalling pathways (Fig. 6a) concurring with our gene ontology analysis (Fig. 4b). For these nodes, several of the connections are already found in the 2i network (Fig. 6b). 2i is a pleiotropic factor that is likely to have several downstream effects and it is unlikely that repressing any one node would be able to replace 2i function completely. Nonetheless, we tested whether the downregulation of these nodes had a functional role in reprogramming, by measuring their ability to replace 2i function at least partially. We chose Egfr: the most highly connected in the AA+2i down network; Igfbp3: key node from the Igf signalling pathway that has not been previously implicated in reprogramming and Egr1: the most downregulated gene in the 2i condition (Supplementary Data 1 and Supplementary Fig. 6a). Remarkably, as compared with AA alone, depletion of any of these transcripts by siRNA-mediated knockdown (Supplementary Fig. 6b) led to up to a fourfold increase in reprogramming efficiency in the simultaneous condition (Fig. 6c) and a greater than fivefold increase in the switch condition (Fig. 6d). Combining knockdowns of all three factors led to cell death. Taken together, these results imply that high expression of the highly connected nodes downregulated by 2i acts as a barrier to reprogramming.

### Lower growth factor signalling activates pluripotency genes

To determine how much of this rewiring represented late stages of reprogramming from fibroblasts and was not exclusive to the state represented in the stalled intermediates, we compared the key nodes from RNA profiling of Thy1^−^SSEA^+^ intermediate cell populations by Polo *et al*.^4^ (Supplementary Fig. 6c). As expected, genes from the pluripotency network were activated late in reprogramming. Remarkably, the highly connected repressed genes were also downregulated in the day 9-to-day 12 transition in fibroblasts, at the same time interval that the pluripotency genes were upregulated (Supplementary Fig. 6c). Thus, reprogramming routes taken by intermediate populations isolated on the basis of their cell surface markers also repress these signalling genes late in the process concomitant with the increase in the pluripotency genes (Supplementary Fig. 6c). These observations provided the intriguing possibility that the downregulation of the repressed nodes may be required for the activation of the pluripotency genes.

**Figure 7:**
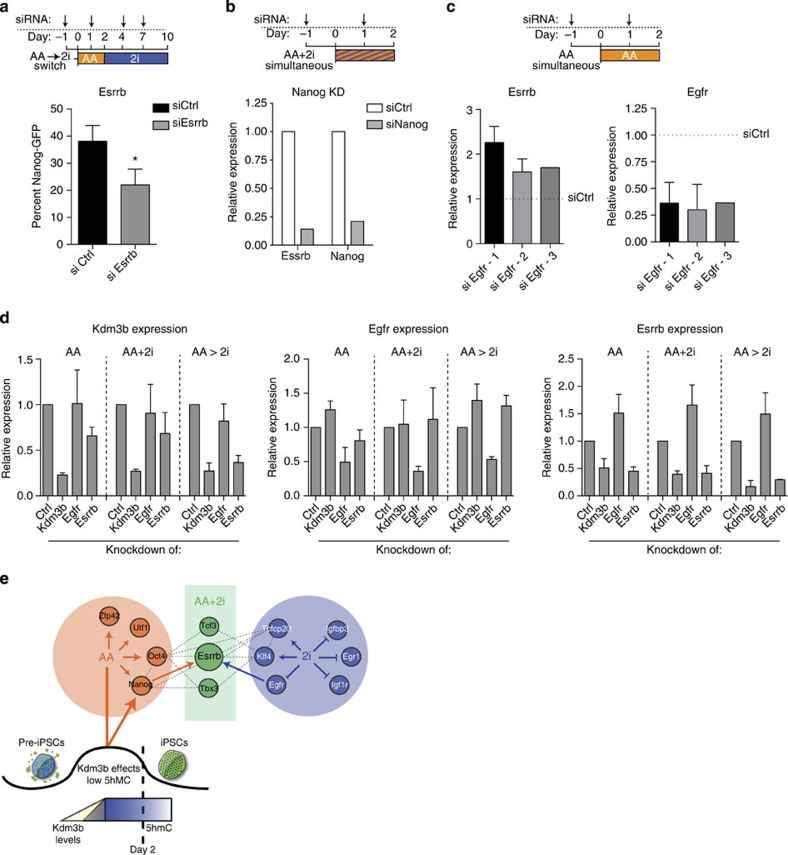
Convergence of AA and 2i effectors to regulate Esrrb level. (**a**) Top panel—Scheme of the experiment: siRNA transfections targeting Esrrb or control (anti-luciferase) were performed on days −1, +1, +4 and +7, after addition of AA on day 0. On day 2, media were switched to that containing 2i alone. Bottom panel—Quantification of Nanog-GFP-positive cells obtained on day 10 of the experiment upon knockdown of Esrrb. Error bars represent standard deviation from four replicates. Asterisk indicates significance *P<0.05 assessed by *t*-test. (**b**) Top panel—Scheme of the experiment: siRNA transfections targeting Nanog or control (anti-luciferase) were performed on days −1 and +1 of simultaneous exposure to AA and 2i, which began on day 0. Bottom panel—Quantification of Esrrb and Nanog expression by quantitative reverse transcription—PCR (qRT–PCR) following 48 h of AA+2i treatment. (**c**) Top panel—Scheme of the experiment: siRNA transfections targeting Egfr or control (anti-luciferase) were performed on days −1, and +1 of exposure to AA, which began on day 0. Bottom panel—Quantification of Esrrb and Egfr expression by qRT–PCR following 48 h of AA treatment. Data from three independent siRNAs indicated as #1,#2 and #3 and five, three or one biological replicates, respectively, are presented. (**d**) Left panel—Kdm3b expression after siRNA transfections targeting control (anti-luciferase), Kdm3b, Egfr and Esrrb were performed on days −1, and +1 of exposure to AA alone (day 2 levels) or AA+2i (day 2 levels) or exposure to AA for 2 days followed by switch to 2i for 2 days (day 4 levels). Middle panel—same as above for Egfr. Right panel—same as above but for Esrrb. Error bars represent standard deviation of two biological replicates. (**e**) Model for action of AA and 2i during reprogramming. Treatment with AA functions through Kdm3b early and Tet-mediated 5hmC increases late in the reprogramming process. In parallel, AA and 2i activate separate key pluripotency genes. 2i also suppresses signalling effectors like Egfr, which in combination with AA can directly regulate a unique cohort of pluripotency genes such as Esrrb. This results in the complete activation of the pluripotency network (dotted line).

To test this hypothesis, we chose to examine the effect on *Esrrb*, as its activation required both the AA and 2i inputs (Fig. 5g). We first validated that Esrrb was a key mediator for AA- and 2i-mediated reprogramming. Depletion of *Esrrb* (Supplementary Fig. 6b) in the switch system led to a significant decrease in reprogramming efficiency (Fig. 7a), confirming its importance in the transition to the iPSC state. Among the AA responsive genes, we depleted *Nanog* (Fig. 7b) and found that *Esrrb* levels correspondingly decreased (Fig. 7b). As we had identified Egfr, Egr1 and Igfbp3 as key nodes that could partially replace 2i function, we tested whether depleting any of these genes had an effect on *Esrrb* expression. Surprisingly, upon knockdown of *Egfr* in the presence of AA, there was up to a twofold upregulation in *Esrrb* levels (Fig. 7c). Thus, high *Egfr* levels play an active role in suppression of pluripotency genes such as *Esrrb*, to block reprogramming which is relieved by 2i.

From our studies, we found that *Kdm3b* (Fig. 3d), *Egfr* (Fig. 6c,d) and *Esrrb* (Fig. 7a) all played important roles in the pre-iPSC to iPSC conversion. We then wanted to test if the expression levels of these key factors were interconnected. Therefore, we depleted *Kdm3b*, *Egfr* and *Esrrb* in the context of AA, AA to 2i and AA+2i conditions. Lowering *Kdm3b* levels significantly downregulated the expression of *Esrrb* in all three conditions, presumably through downregulation of *Nanog* (Fig. 7b,d). Conversely, depletion of *Egfr* strikingly increased *Esrrb* levels in the AA alone condition (Fig. 7d and Supplementary Fig. 7b). It is important to note here that similar to results obtained from the RNA-Seq (Supplementary Fig. 6c) and time-course experiments (Supplementary Fig. 6a), we found that especially in the presence of 2i, *Egfr* levels were lowered (Supplementary Fig. 7a). Therefore, it was not possible to achieve a much further reduction in *Egfr* levels by siRNA-mediated knockdown in the AA+2i conditions (Supplementary Fig. 7a). The levels of *Egfr* and *Kdm3b* were not reciprocally regulated. Finally, depletion of *Esrrb* did not affect the levels of *Egfr*.

## Discussion

Using our two-component system, we are able to gain a clearer picture of how the pluripotency network is rewired during the late stages of reprogramming as represented by the pre-iPSC to iPSC transition. Our results demonstrate that as the iPSC state is reached, pluripotency genes are activated in three separate modules- responsive to chromatin changes, responsive to signal inhibition or requiring both activities (Fig. 7e).

H3K9 methylation and DNA methylation are repressive marks that often occur together and are dependent on each other^40^. From our results, it is clear that the H3K9 demethylase Kdm3b is essential to the early AA phase, when global levels of also 5hmC increase. Several of the upregulated genes also occurred at contiguous genomic locations: *Nanog, Dppa3, Gdf3, Apobec1* (chr 6); *Dppa4* and *Dppa2* (chr 19); *Dppa5a* and *Ooep* (chr 9). Since H3K9me2 occurs in large genomic blocks^41^, this result suggests that AA exposure may allow the spreading of the erasure of this mark. Interestingly, depletion of Kdm3b was also found to diminish human reprogramming^42^ in an unbiased screen, suggesting conservation of mechanism of action between these systems. In contrast to ESCs, during the late stage of reprogramming, exposure to AA but not 2i increases 5hmC levels. Although a recent report suggested that Tet function is required for the mesenchymal-to-epithelial transition^34^, our results support the requirement for Tet function during the late stages of pluripotency^35^.

Nanog, which has a central role in reprogramming, is highly connected in the AA+2i upregulated network. Two of its connected edges, *Utf1* and *Dppa2*, are upregulated in the AA or AA+2i conditions by day 2. These genes have been implicated as better prospective markers of reprogramming in a population^43^, and also represent the stabilization phase of the reprogramming process^44^. However, their upregulation was insufficient to reach high levels of reprogramming efficiency, highlighting the importance of the 2i contribution to reaching the iPSC state. Pluripotency genes that are activated following exposure to 2i include *Klf4* and *Tcfcp2l1*. *Tcfcp2l1* has recently been described as a key player in responsiveness to LIF in ESCs^45^,^46^, which is a necessary component in our medium for successful reprogramming. Of note neither of these genes is significantly changed in expression when ESCs are switched from growth in serum to growth in 2i^23^.

Growth factor signalling inhibition can maintain ESCs in the pluripotent state by preventing differentiation cues^20^. Similarly, we find that in the late stage of reprogramming repressing highly connected downregulated genes could replace 2i function, albeit partially, by enhancing activation of pluripotency genes. This suggests that other genes that are activated by 2i such as the Wnt-related *Axin2* likely play a role in establishing pluripotency. Interestingly, it was recently shown that *Egr1* was also an impediment for human reprogramming^47^. Together these results point to specific growth factor signalling as a common barrier to reprogramming.

Several reprogramming strategies employ components of 2i as part of the media. In an interesting parallel to our system, in chemically derived iPSCs^48^, the addition of 2i is the very last step and is preceded by the addition of the chemical DZNep, a histone methyltransferase inhibitor. Although we did not observe any changes in Mbd3 levels, the deterministic conversion of somatic cells to iPSCs in the absence of the chromatin regulator Mbd3 (ref. 49) occurs only in the presence of 2i. In addition, several protocols to recover and support naïve human reprogramming also utilize 2i^50^,^51^,^52^. While this paper was under review, two recent publications reported that AA in combination with the GSK inhibitor alone^14^ or in addition to a TGF-β inhibitor^13^ resulted in high efficiency of reprogramming from different somatic precursor cell types and moderate levels from MEF reprogramming populations. Significantly, similar to our results (Fig. 1), MEF reprogramming was further enhanced by the addition of the MEK inhibitor^13^. The AA+GSKi effects were attributed to the conversion of early intermediates that have not yet gained the SSEA cell surface marker. As our pre-iPSCs already express the SSEA marker, these results suggest that the contributions of each of the 2i components may differ at early and late stages of reprogramming^42^. Our data complement these studies and we have now begun to delineate the molecular mechanisms that are likely to contribute to all these systems.

## Methods

### Cell culture

Male and female MEFs were isolated from E13.5 embryos from time-mated mice. Fibroblasts and astrocytes, used for deriving pre-iPSCs, were heterozygous for R26 rtTA and contained the four reprogramming factors in a single cassette with one allele in a OKSM^28^ and the other in a OSKM^29^ configuration. MEFs used for sorting experiment and second replicate of adult fibroblasts were heterozygous for R26 rtTA and homozygous for the OKSM allele. MEFs were maintained in Knockout DMEM (Life Technologies) supplemented with 10% fetal bovine serum (FBS), penicillin–streptomycin, Glutamax and non-essential amino acids (NEAA). For tail-tip and dermal fibroblast isolation, tail skin was placed dermal-side down in Trypsin-EDTA overnight at 4 °C. The dermis was separated from the epidermis and dermal tissue placed on gelatin-coated 6-cm plates under coverslips in fibroblast growth medium. For ear fibroblast isolation, ear pieces were placed in trypsin-EDTA at 37 °C for 1 h with regular vortexing and then placed under coverslips on gelatin-coated plates in fibroblast growth medium. Astrocytes were isolated from d4 post-natal pups. Cortices were mechanically dissociated and plated on 10-cm tissue culture-treated plates in DMEM+10% FBS supplemented with penicillin (50 U ml^−1^) streptomycin (50 μg ml^−1^), Glutamax (1 × ) and NEAA (1 × ) for 4 days then FBS was reduced to 1%.

### Reprogramming

Reprogramming was performed in ESC media: Knockout DMEM (Life Technologies) supplemented with 15% FBS, penicillin–streptomycin, Glutamax, NEAA and LIF. Reprogramming was initiated with the addition of 2 μg ml^−1^ of dox for 3 days followed by treatment of AA (Sigma; 50 μg ml^−1^) and 2i: 1 μM PD-0325901 (Stemgent) and 3 μM CHIR-99021 (Stemgent) with dox, at 3 day intervals. Pre-iPSC lines were derived as previously described in Sridharan *et al*.^6^ Briefly, *Nanog*-GFP reporter MEFs were transduced with pMX retroviruses encoding Oct4, Sox2, c-Myc and Klf4 in ESC media. Colonies with ESC-like morphology and without GFP expression were picked and maintained for several generations in ESC media. Astrocyte pre-iPSCs were derived from mice that did not have the Nanog-GFP reporter and were isolated by immunostaining for Nanog. Pre-iPSCs conversion was initiated with 2 × 10^5^ cells per well in gelatinized six-well plates. AA- or 2i-containing ESC media were replaced at 3-day intervals or 2-day intervals for temporal experiments (Fig. 2). Cells were passaged on day 4, harvested on day 10 for analysis by flow cytometry or counts of immunostained Nanog-positive colonies. Experiments were performed in biological triplicates. Media were supplemented with 3 μM 5-Azacytidine (Acros Organics) in indicated experiments.

### Flow cytometry

For FACS analysis, cells were harvested with trypsin into single-cell suspensions and analysed on a FACS Canto II (BD Biosciences) or Accuri. Data were analysed using the FlowJo software (TreeStar). SSEA-positive and Thy-negative MEFs were sorted on BD FACS ARIA III using PE-conjugated Thy1.2 (eBioscience #12-0903-81) and Alexa Fluor 647-conjugated SSEA-1 (BioLegend #125608) antibodies.

### *In vitro* differentiation

Cells were plated at 2 × 10^4^ cells per cm^2^ on gelatin-coated six-well plates in ESC media overnight. Media were changed to chemically defined media^53^ with varying growth factors or small molecules for directed mesoderm ((BMP4 (20 ng ml^−1^), Activin A (2 ng ml^−1^) and Wnt3a (3 ng ml^−1^); Gata2, endoderm (phorbol myristic acetate (TPA) 50 nM; Gata4, Sox17 and FoxA2) and ectoderm (all *trans*-retinoic acid; 10^−5^ M)) differentiation (Pax6). Cells were differentiated in FBS-containing media (IMDM+15% FBS, penicillin (50 U ml^−1^), streptomycin (50 μg ml^−1^), Glutamax (1 × ) and 1-thioglycerol (4.5 × 10^−4^ M)). Differentiations were analysed on day 7 by reverse transcription–PCR.

### Teratoma formation

Teratoma formation was performed essentially as in Yu *et al*.^54^ Briefly, C.B-17/IcrHsd-PrkdcscidLystbg (6- to 7-week-old females) were purchased from Harlan Laboratories.

Approximately 1 million cells in 200 μl of each iPSC clone were injected into intramuscular hind leg of three mice. Teratomas were removed 2.5 weeks after injection before exceeding 2 cm in diameter, rinsed in PBS and placed in 10% formalin for histology performed in the UW-Veterinary school.

### 5hmc/5mC dot blots

5hmC and 5mC dot blots were performed essentially as in Szulwach *et al*.^55^ Briefly, cells were lysed overnight and DNA was precipitated, and quantified by QuBit. Samples were cross-linked onto a nitrocellulose membrane and blotted with 5hmC (1:10,000) or 5mC (1:5,000) (Active Motif 39770 and 61228, respectively) overnight at 4 °C and detected with chemiluminiscence.

### Immunofluorescence

Immunofluorescence was performed essentially as in Sridharan *et al*.^6^ Antibodies used were Nanog (1:100; CosmoBio Co RCAB0002P-F), Esrrb (1:200; R&D Biosystems H6075), Pecam1 (1:100; BD Bioscience 557355).

### siRNA transfection

Sets of four different siRNAs were purchased from Dharmacon and transfected using lipofectamine–RNAi max (Life Technologies) according to the manufacturer’s instructions. Of the set of four siRNAs, the one producing the most efficient knockdown was used in reprogramming experiments at a final concentration of 20–50 μM: Tet1 MU-062861-11-0002, Tet2 MU-058965-07-0002, Tet3 MU-054156-11-0002, Kdm3a MU-056510-01-0002, Kdm3b MU-065381-02-0002, Kdm3b J-065381-06-0002, Kdm3c MU-062872-04-0002, Ehmt1 LU-059041-01-003, Ehmt2 MU053728-003, Egfr MU-040411-01-0002, Egfr MU-040411-02-0002, Egfr MU-040411-04-0002, Egr1 MU-040286-03-0002, Igfbp3 MU-046554-01-0002, Igfbp3 MU-046554-04-0002, Nanog MU-057004-02-0002, Essrb MU-059177-01-0002, Essrb MU-059177-02-0002 or non-targeting Luciferase control D-001210-02.

### Quantitative reverse transcription–PCR

To test knockdown efficiency during reprogramming or transcript levels after *in vitro* differentiation, RNA was harvested 2 days after transfection and processed with Qiagen (#74104) or Zymo research kits (R2052) and quantitative real-time reverse transcription—–PCR was performed with gene-specific primers listed below. Reprogrammed clones were tested for lack of exogenous expression and increased endogenous expression of reprogramming factors.

List of primers used:


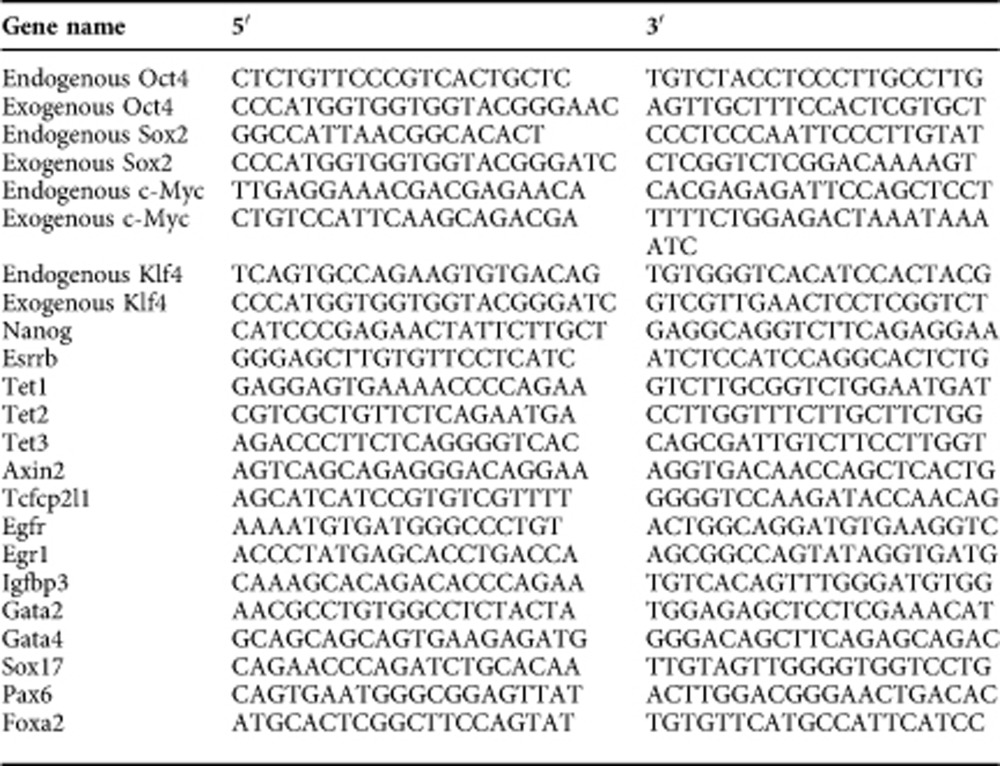


### Expression profiling

RNA was extracted from cells treated with DMSO, AA, 2i or AA+2i using Qiagen RNeasy Mini Kit (#74104) and quantified using Qubit. Single-end cDNA library was constructed using TruSeq RNA Sample Preparation kit RS-122-2001 (Illumina) according to the manufacturer’s instructions. Samples were multiplexed and sequenced on a HiC2000 at the University of Wisconsin—Biotechnology Center. Experiments were performed in biological triplicate.

### RNA-Seq analysis and computational analysis

Greater than 25 million 100 bp reads were sequenced per sample. Sequence reads were trimmed (first 10 bp were removed) in order to remove GC bias at the beginning of each read. Six-basepair Illumina adapter sequences were clipped and processed reads were filtered to only retain high-quality sequences (>30 quality score determined by Fastx). Processing and filtering of sequence reads were performed using Fastx Toolkit (Fastq Trimmer- Trimming, Fastq Clipper—Adapter removal, and Fastq Quality Filter—Exclusion of low quality reads). Accepted sequences were aligned to mouse reference genome (mm9) constructed to include only annotated genes (that is, NM_ RefSeqs) by Bowtie2 alignment with a mismatch per seed set to 2 and seed length set to 28. Gene expression of aligned reads was quantified using RSEM with a forward probability of 0.5 (ref. 56). As the three biological replicates had a high correlation, the median of the data is presented in Supplementary Data 1. DE was calculated using EB-Seq programme^38^, which uses a Bayes approach to calculate the distribution of expression. This model assigns posterior probability for individual genes expression pattern, so that thresholds can be used to reduce false discovery rates. Genes were considered differentially expressed with a posterior probability greater than 0.95. Data have been deposited in the GEO database, accession code GSE58836.

K-means clustering was performed using Gene Cluster 3.0 and visualized by Java TreeView. Identification of enriched functional annotation for individual clusters were determined by DAVID Bioinformatics Resources^57^. Furthermore, Genes associated with Gene Ontology term Stem Cell Maintenance (GO:0019827) with additional information from Golipour *et al*.^44^ were overlapped with treatment-specific DE genes. DE genes were compared with published iPSC and pre-iPSC data sets from Sridharan *et al*.^30^

### Network analysis

Networks were generated by overlaying DE genes that were further induced (UP) or repressed (DOWN) by twofolds in AA, 2i or AA+2i conditions on to the STRING database^39^. The STRING database (http://string.embl.de/newstring_cgi/show_input_page.pl?UserId=l9EpVn3Ktnei&sessionId=ojR_97V0pL3q) is a collection of known and predicted protein–protein interactions, which quantitatively collates data from (i) genomic contexts, (ii) high-throughput experiments including proteomic data and ChIP-Seq data, (iii) co-expression data from RNA-Seq and microarray data and (iv) literature parsing of full publications. The database contains information on almost 5 million proteins from 1,133 organisms. Depending on the evidence from gene fusion, co-occurrence, co-expression, direct experimental evidence and text mining each putative interaction between two proteins is given a confidence score. We downloaded the network (http://string-db.org/newstring_download/protein.links.detailed.v9.1/10090.protein.links.detailed.v9.1.txt.gz) and retained any edge that had a high confidence of ≥0.5.

There were a total of six gene sets, two for each direction of expression for each of the three conditions. A network was generated for each gene set by including only those edges that connected two genes in the same gene set. To determine whether a network generated from a specific gene set was enriched for interactions, a *z*-score was computed for the number of edges in the gene set to the number of edges associated with random gene sets of the same size. The z-score was computed by estimating the mean and standard deviation of the number of edges associated with ten random gene sets. The more positive a *z*-score the more enriched the gene set of interest is enriched for interactions.

To examine whether genes that are commonly induced in 2i and AA+2i (or AA or AA+2i), and are more connected to genes that are induced only in AA+2i, a separate *z*-score was calculated. For genes that were induced only in 2i and not in AA+2i, the number of edges that connect a gene in the common set to genes that are induced only in 2i (that is, a bi-partite graph) was computed. Next random gene sets of the same size as the genes induced only in 2i were generated, and a mean and standard deviation of edges that connect the common gene set and these random sets was computed. Using this mean and standard deviation, we computed a *z*-score that assessed the number of interactions connecting the genes induced in both 2i and AA+2i to genes induced only in 2i. The same procedure was repeated for all other overlaps. Networks were visualized using Cytoscape^58^.

### Statistical analysis

Significance was calculated using the two-tail paired *t*-test function in Graphpad Prism for Figs 4 and 5, and Wilcoxon non-parametric test (Fig. 6b) in the R statistical package.

## Author contributions

R.S. conceived the project and wrote the manuscript; K.A.T.^58^ prepared the figures; R.S. and K.A.T. interpreted the results; K.A.T., S.A.J., Z.P.G.O., N.Z.Z. and R.S. performed the experiments and analysed the data. N.L. performed the EB-Seq analysis under the guidance of C.K.. S.R. performed the network analysis and wrote the corresponding portions of the manuscript.

## Additional information

**How to cite this article:** Tran, K. A. *et al*. Collaborative rewiring of the pluripotency network by chromatin and signalling modulating pathways. *Nat. Commun.* 6:6188 doi: 10.1038/ncomms7188 (2015).

**Accession codes:** Gene expression data have been deposited in the GEO database, accession code GSE58836.

## Supplementary Material

Figures and Supplementary References Supplementary Figures 1-7 and Supplementary Reference

Supplementary Data 1 Detailed gene expression data for individual treatments. Sheet 1 contains absolute transcript per million (TPM) values for each replicate for all genes. Sheet 2 contains list of differentially expressed (DE) genes upregulated compared to DMSO determined by EB-Seq using FDR 0.05. Sheet 3 contains list of DE downregulated genes determined by EB-Seq using FDR 0.05. For DE genes, posterior probability and median TPM values for individual treatments is reported.

Supplementary Data 2 Information on networks constructed using the STRING database set at a confidence level of 0.5 for individual treatments. Sheet 1 contains a list of downregulated or upregulated nodes and their connections with treatment of AA, 2i or both. Sheet 2 lists either AA or 2i nodes that overlapped with the AA+2i upregulated and downregulated networks. For overlapping genes, numbers of connections with each treatment is reported.
