# Supplementary material for: Collaborative rewiring of the pluripotency network by chromatin and signalling modulating pathways
Source: Nat Commun. 2015 Feb 4;6:6188. doi: 10.1038/ncomms7188 (PMC4347202; doi:10.1038/ncomms7188)
Supplement: Figures and Supplementary References — Supplementary Figures 1-7 and Supplementary Reference [file ncomms7188-s1.pdf]

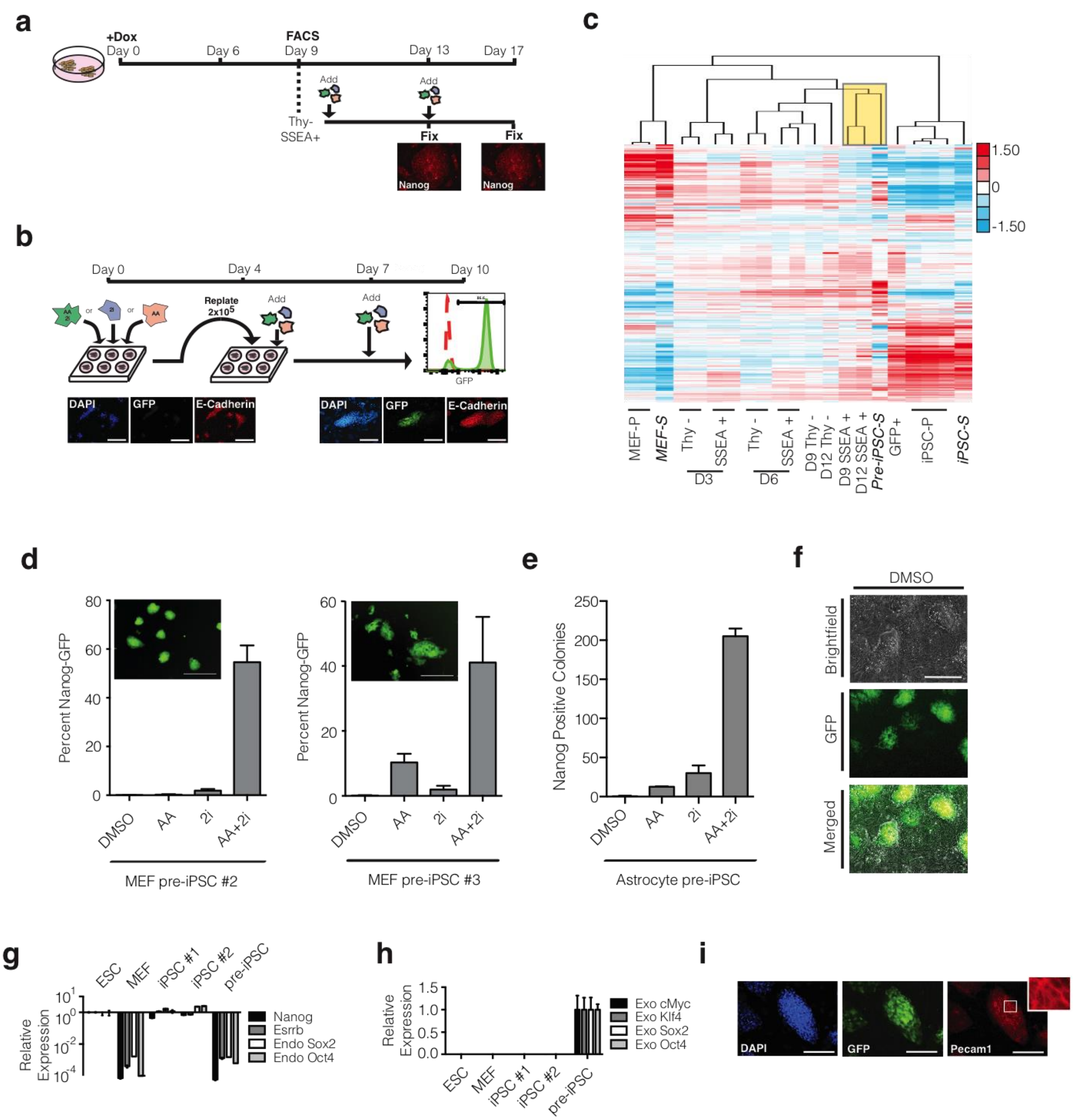

Supplementary Figure 1: AA+2i synergistically promotes pre-iPSC to iPSC conversion

a) Scheme of experiment (Related to Figure 1d): MEFs were treated with Doxcycline at Day 0 to induce the reprogramming factors Oct4, Sox2, Klf4, and c-Myc (OSKM). Cells were sorted for Thy1-SSEA1+ population on Day 9, which was treated with DMSO, AA, 2i, or AA+2i containing media. Media with treatment was replaced every three days and Nanog positive colonies were counted on Day 13 or 17. **b)** Scheme of experiment (Related to Figure 1e): pre-iPSCs receive AA and/or 2i or control DMSO treatment on day 0, day 4 and day 7, followed by flow cytometry

analysis for Nanog-GFP positive cells on day 10. Cultures were split on day 4 to account for expansion of DMSO treated cells. Bottom panel – Immunofluorescence detection of E-cadherin (red) and Nanog-GFP (green) and DAPI – left on day 0 and right on day 10. Scale bar = 100 μm. **c)** Hierarchical clustering of gene expression from MEFs, iPSCs and pre-iPSCs from Sridharan et. al. 2009 – labeled with the suffix –“S” and sorted intermediate populations from MEF reprogramming from Polo et al., 2012- labeled with the suffix-"P". Data from each publication was normalized independently and then merged. Red represents greater than mean and Blue lower than mean value for each gene. pre-iPSCs clustered with Day 9 and Day12 Thy1-SSEA1+ populations during MEF reprogramming- Highlighted in yellow. **d)** Bar graph representing Nanog-GFP reporter expression for two independent MEF derived pre-iPSC lines by flow cytometry. **e)** Astrocyte derived pre-iPSC by Nanog immunofluorescence upon exposure to AA and/or 2i or DMSO. Inset: Representative image of Nanog-GFP^{+} colonies after 10 days of exposure to AA+2i. Scale bar = 250μm. **f)** Top- Brightfield image of iPSC colonies obtained from AA+2i reprogrammed pre-iPSC maintained on MEFs in DMSO. Center –Nanog-GFP reporter expression. Bottom – Merged brightfield and fluorescence images. **g)** RT-PCR confirming expression of pluripotency markers Nanog and Esrrb, and activation of endogenous Oct4 and Sox2 loci. Error bars indicate standard deviation of three technical replicates from one representative experiment with ESC levels set to 1. iPSC #1 and #2 are two independent clones obtained after exposure to AA+2i. **h)** qRT-PCR confirming inactivation of exogenous retroviral expression of reprogramming factors Oct4, Sox2, cMyc and Klf4. Error bars indicate standard deviation of three technical replicates from one representative experiment with pre-iPSC levels set to 1. iPSC #1 and #2 are two independent clones obtained after exposure to AA+2i. **i)** Immunofluorescence images of AA+2i derived iPSC clone for Pecam1 (red), DAPI (blue) and Nanog-GFP (green). Inset: magnification of boxed region. Scale bar = 100μm.

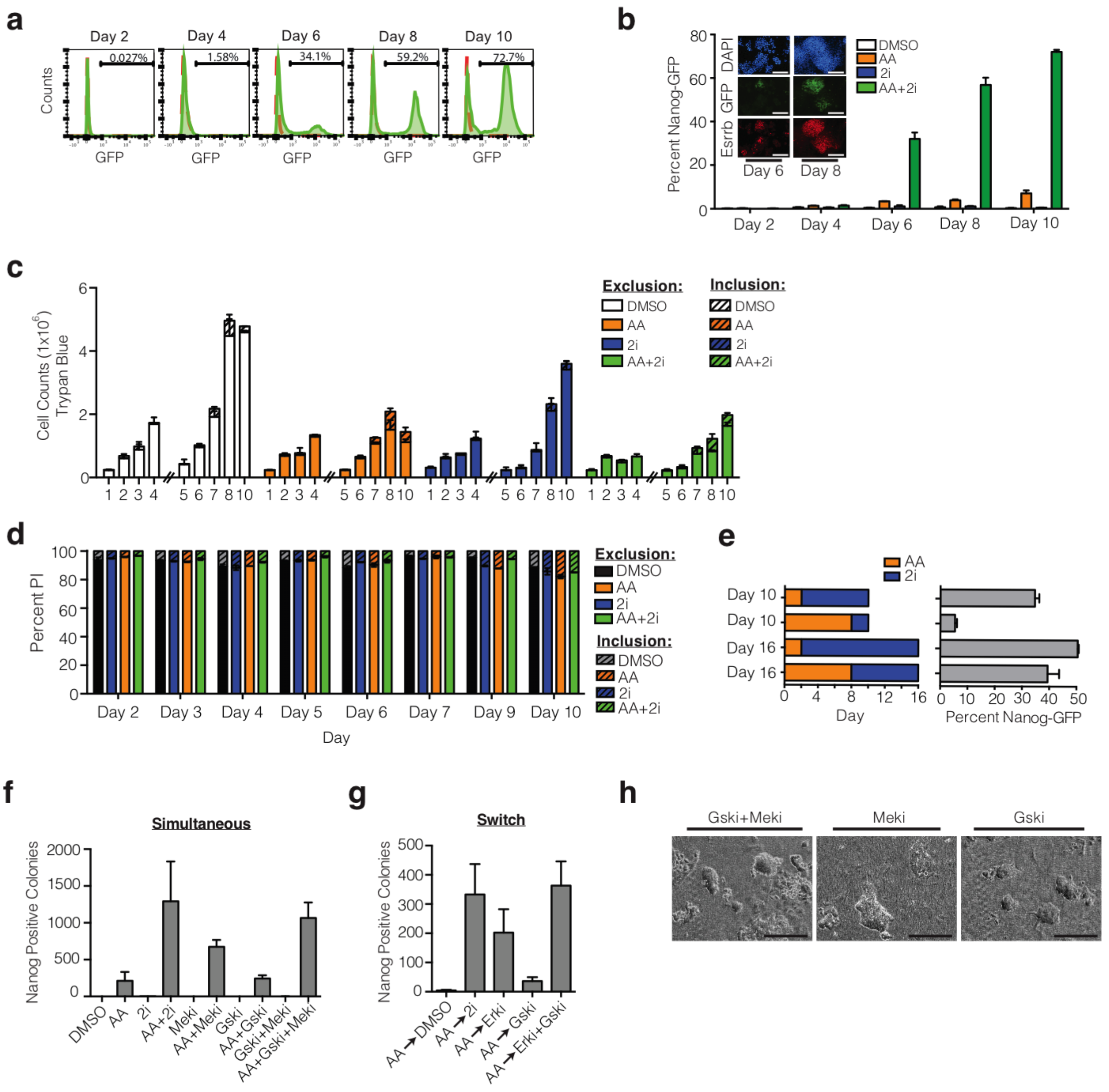

Supplementary Figure 2: Majority of pre-iPSCs convert to the iPSC state.

a) Flow cytometry profiles showing time course of emergence of Nanog-GFP positive cells upon exposure to DMSO (dashed red line) and AA+2i conditions (solid green line). **b)** Quantification of flow cytometry data from Supp Fig 2a. Error bars indicate standard deviation of two technical replicates from one representative experiment. Inset - Representative immunofluorescence images of reprogramming cultures on indicated days detecting Esrrb in red, Nanog-GFP in green, and DAPI in blue. Scale bar = 100μm. **c)** Analysis of live and dead cells upon exposure to DMSO, AA, 2i or AA+2i. Counts of cells on each day of the experiment after exposure to indicated treatments measured with trypan blue exclusion. Error bars represent standard

deviation of two technical replicates from one representative experiment. Culture was split on day 4. **d)** Live cells were measured by propidium iodide exclusion (solid bar) and dead cells by internalization (hatched bar). Error bars represent standard deviation of two technical replicates from one representative experiment. **e)** Longer 2i exposure results in higher percentage conversion to Nanog-GFP positive cells. Left Panel – Scheme of experiment: Cells were treated with AA (orange) before switch to 2i (blue) containing media. Experiments were analyzed on day 10 or day 16 of culture. Right Panel – Summary of flow cytometry results analyzing Nanog-GFP positive cells on day 10 and day 16 of reprogramming. Error bars indicate standard deviation of two technical replicates from one representative experiment. **f)** Nanog positive colony counts on day 10 after simultaneous experiment in the presence of AA and combined or individual components of 2i. Error bars represent standard deviation of four replicates. **g)** Nanog positive colony counts on day 10 after switch experiment in the presence of AA or each inhibitor for 2 days followed by signaling inhibitors or AA for 8 days respectively. Error bars represent standard deviation of four replicates. **h)** Representative images of reprogramming Day 7 cultures in the presence of Meki alone, Gski alone or Meki+Gski.

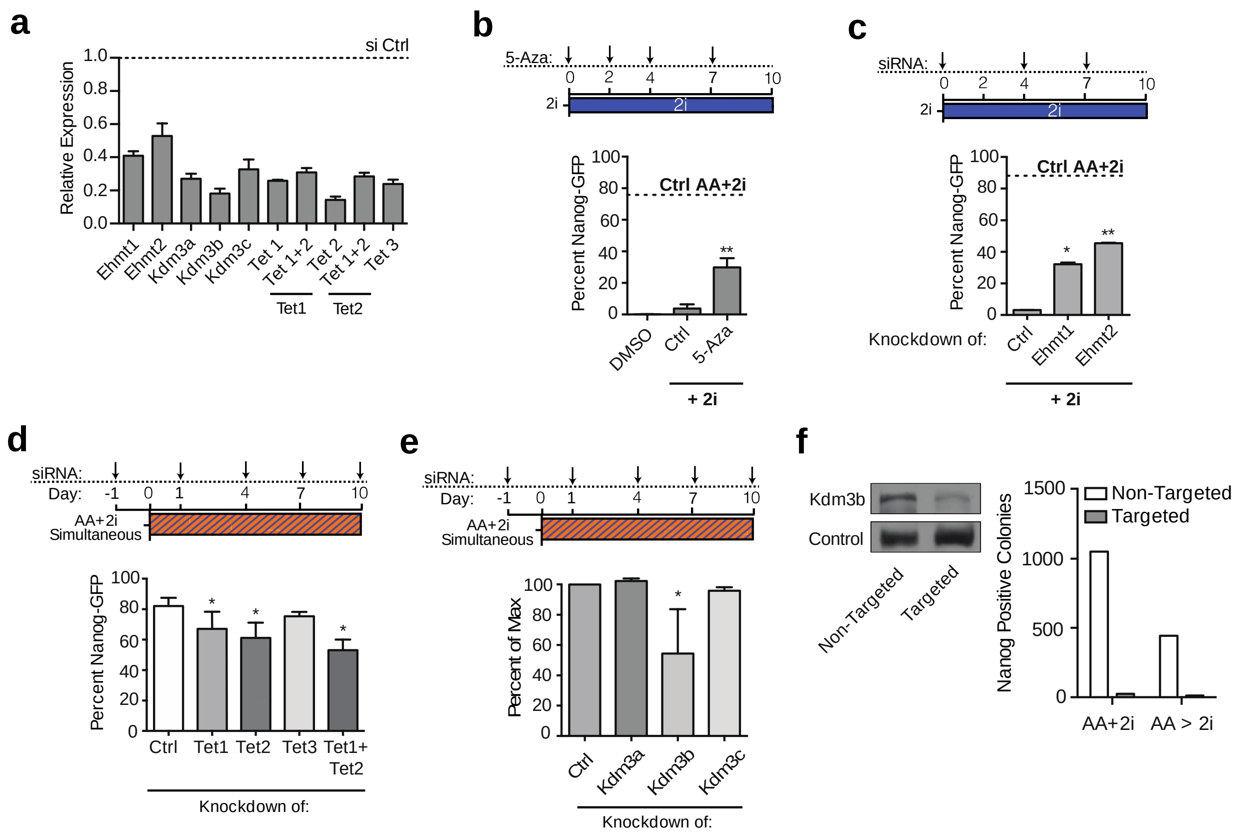

Supplementary Figure 3: pre-iPSC conversion require Kdm3b and Tet1/2

a) qRT-PCR confirming knockdown of Ehmt1, Ehmt2, Kdm3a, Kdm3b, Kdm3c, Tet1, Tet2 and Tet3 when transfected individually or combined (Tet1 + Tet2) relative to control (anti-luciferase) set to 1, at 48 hours. **b)** Top panel –Media containing DMSO, 2i or 2i+5-Azacytidine was added at day 0, day 2, day 4 and day 7. Conversion to Nanog-GFP positive cells was assayed at day 10. Bottom panel – Bar graph of pre-iPSC conversion to Nanog-GFP positive cells following treatment with DMSO, 2i or 2i+5-Azacytidine. Dotted line shows conversion with AA+2i. Error bars represent standard deviation of four replicates **= $p<0.05$ by t-test. **c)** Top panel - siRNA transfections targeting H3K9me2 histone methyltransferases or control (anti-luc) were performed on days 0, 4 and 7 in the presence of 2i alone. Bottom panel - Quantification of Nanog-GFP positive cells obtained on Day 10. Dotted line = AA+2i level. Error bars represent standard deviation from four replicates. * = $p< 0.05$ ** = $p < 0.01$ by t-test. **d)** Top panel - siRNA transfections targeting specific Tet enzymes or control were performed on days -1, 1, 4 and 7 of simultaneous exposure to AA and 2i which began on day 0. Bottom panel – Quantification of Nanog-GFP positive cells obtained on Day 10 Tet1, Tet2, Tet3 or combined Tet1 and Tet2 conditions or anti-luc control. Error bars represent standard deviation of three biological replicates. * = $p< 0.05$ by t-test. **e)** Top panel - siRNA transfections targeting H3K9me2

demethylases or control (anti-luciferase) were performed on days -1, 1, 4 and 7 of simultaneous exposure to AA and 2i which began on day 0. Bottom panel - Quantification of Nanog-GFP positive cells obtained on Day 10. Error bars represent standard deviation from three biological replicates. * = $p < 0.05$ by t-test. **f)** Left-Western blot for Kdm3b in Crispr-Kdm3b targeted and non-targeted pre-iPSC line Right- Count of Nanog-GFP colonies obtained from the non-targeted and targeted pre-iPSC clones in simultaneous and switch conditions.

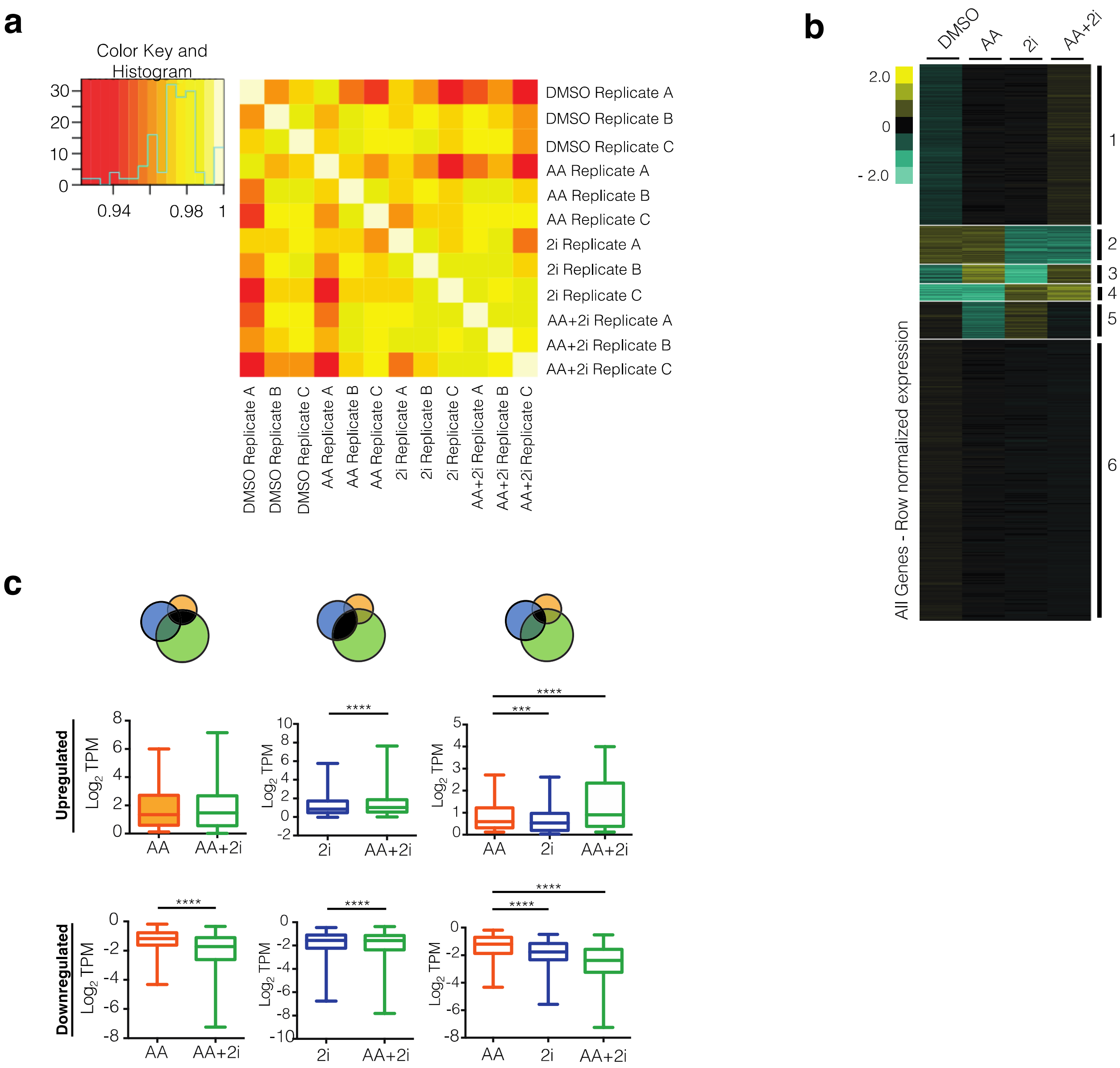

Supplementary Figure 4: AA and 2i have divergent transcriptional responses

a) Spearman co-efficient correlation for three RNA-Seq biological replicates (A, B and C) for DMSO, AA, 2i and AA+2i are displayed. Note the high correlation within each treatment group across the three biological replicates. **b)** K-means clustering into six clusters for expression values (in transcripts per million) normalized across each row shows that certain AA and 2i transcriptional responses are opposite of each other, for example clusters 2, 3, 4 and 5. The absolute expression for each gene (row) was normalized based on the median across all four conditions. Yellow represents greater than median and blue represents lower than median value for that particular gene. **c)** Box plots showing Log_2 normalized gene expression of overlaps between differentially expressed genes in the three different conditions- Orange = AA alone,

Blue = 2i alone and Green = AA+2i. Source of overlap for each plot is highlighted in the Venn diagram in black. Gene expression was measured in transcripts per million (TPM). Error bars represent deviation from the median, significance was calculated using Wilcoxon test: ****p>0.0001, *** p>0.001.

a

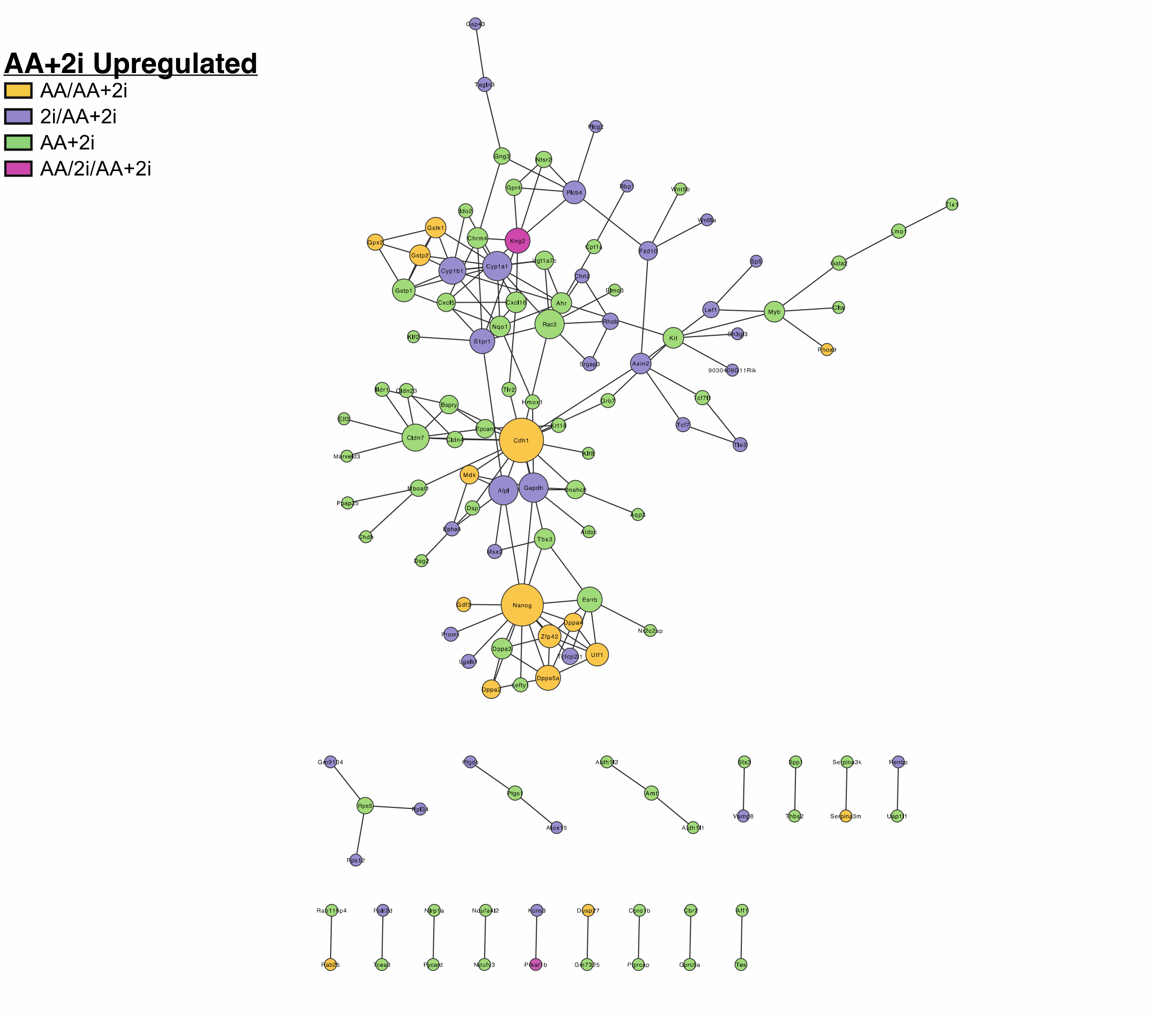

b

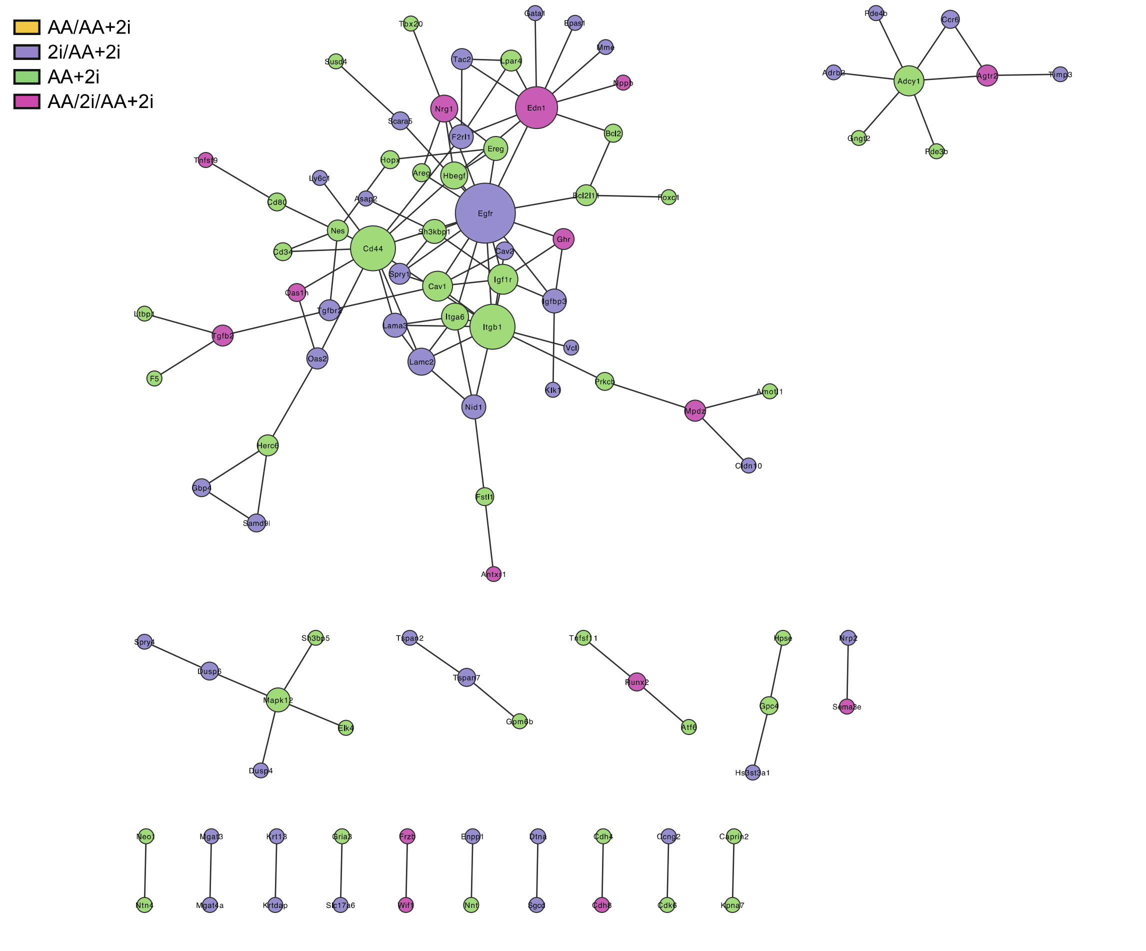

c

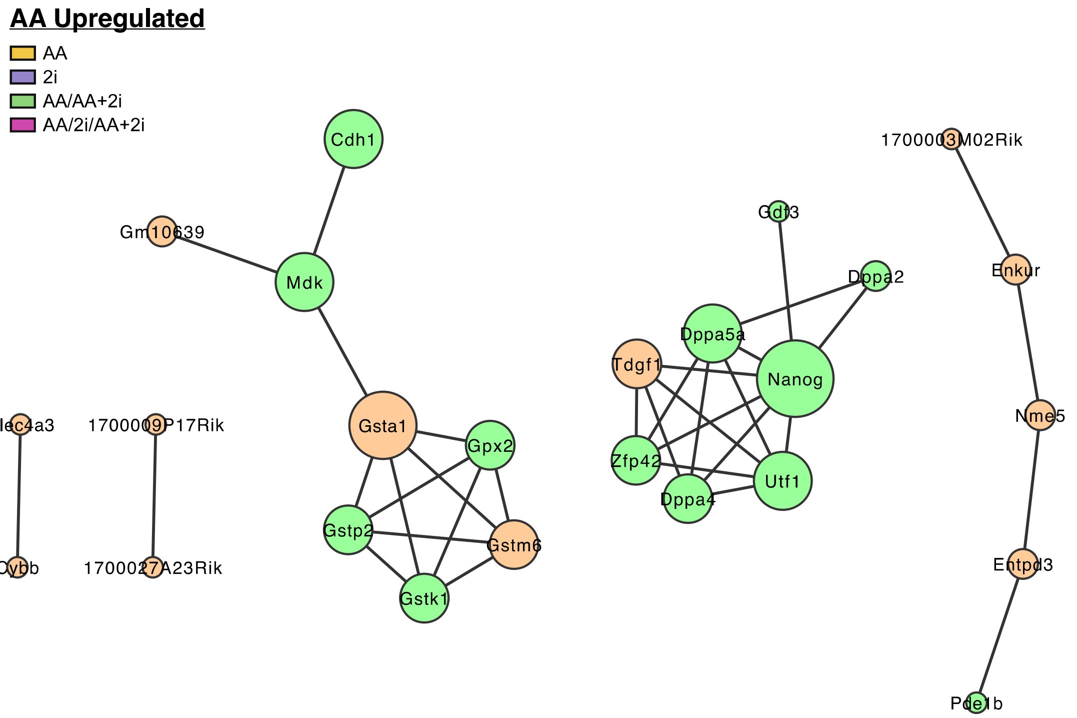

AA Upregulated
AA
2i
AA/AA+2i
AA/2i/AA+2i
Cdh1
Gm10639
Mdk
Gsta1
Gpx2
Gstp2
Gstm6
Gstk1
Clec4a3
Cybb
1700009P17Rik
1700027A23Rik
Gdf3
Dppa2
Dppa5a
Tdgf1
Nanog
Zfp42
Dppa4
Utf1
1700003M02Rik
Enkur
Nme5
Entpd3
Pde1b

d

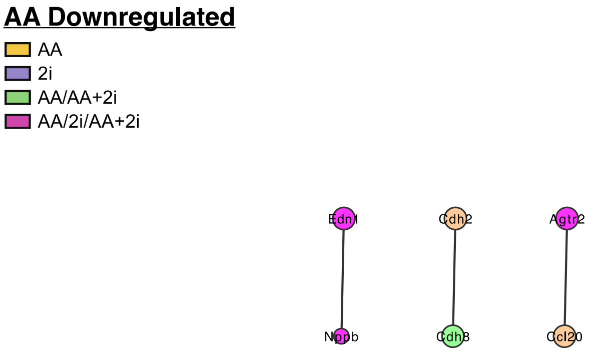

AA Downregulated
AA
2i
AA/AA+2i
AA/2i/AA+2i
Edn1
Nppb
Cdh2
Cdh8
Agtr2
Ccl20

e

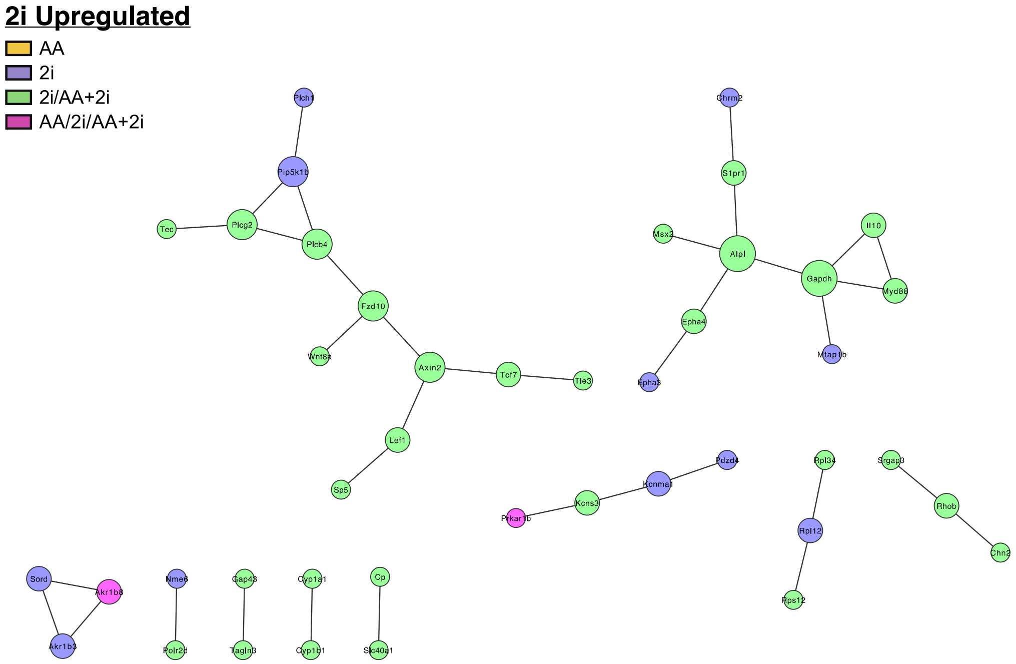
2i Upregulated
AA
2i
2i/AA+2i
AA/2i/AA+2i
Plch1
Pip5k1b
Plcg2
Tec
Plcb4
Fzd10
Wnt8a
Axin2
Tcf7
Tle3
Lef1
Sp5
Chrm2
S1pr1
Msx2
Alpl
Il10
Gapdh
Myd88
Epha4
Epha3
Mtap1b
Pdzd4
Kcnma1
Kcns3
Prkar1b
Rpl34
Rpl12
Rps12
Srgap3
Rhob
Chn2
Sord
Akr1b8
Akr1b3
Nme6
Polr2d
Gap43
Tagln3
Cyp1a1
Cyp1b1
Cp
Slc40a1

f

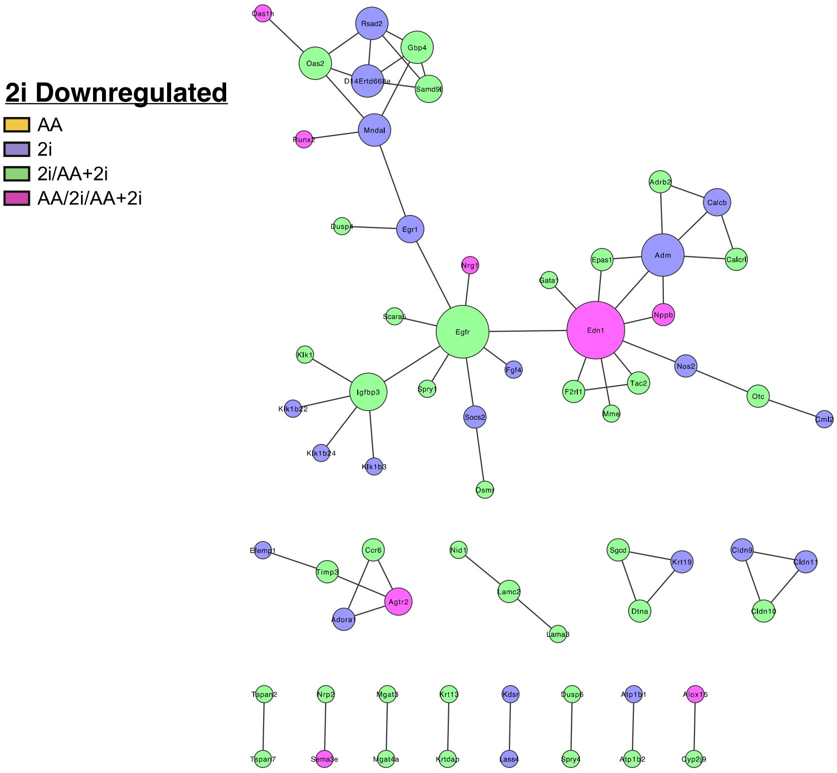
2i Downregulated
AA
2i
2i/AA+2i
AA/2i/AA+2i
Oas1h
Rsad2
Gbp4
Oas2
D14Ertd668e
Samd9l
Runx2
Mndal
Dusp1
Egr1
Nrg1
Scara5
Egfr
Klk1
Igfbp3
Spry1
Fgf4
Klk1b22
Klk1b24
Klk1b3
Socs2
Osmr
Adrb2
Calcb
Epas1
Adm
Calcrl
Gata1
Nppb
Edn1
Nos2
Otc
Cmtd
F2rl1
Tac2
Mme
Efemp1
Ccr6
Timp3
Agtr2
Adora1
Nid1
Lamc2
Lama3
Sgcd
Krt19
Dtna
Cldn9
Cldn11
Cldn10
Tspan2
Tspan7
Nrp2
Sema3e
Mgat3
Mgat4a
Krt13
Krtdap
Kdsr
Lass4
Dusp6
Spry4
Atp1b1
Atp1b2
Alox15
Cyp2j9

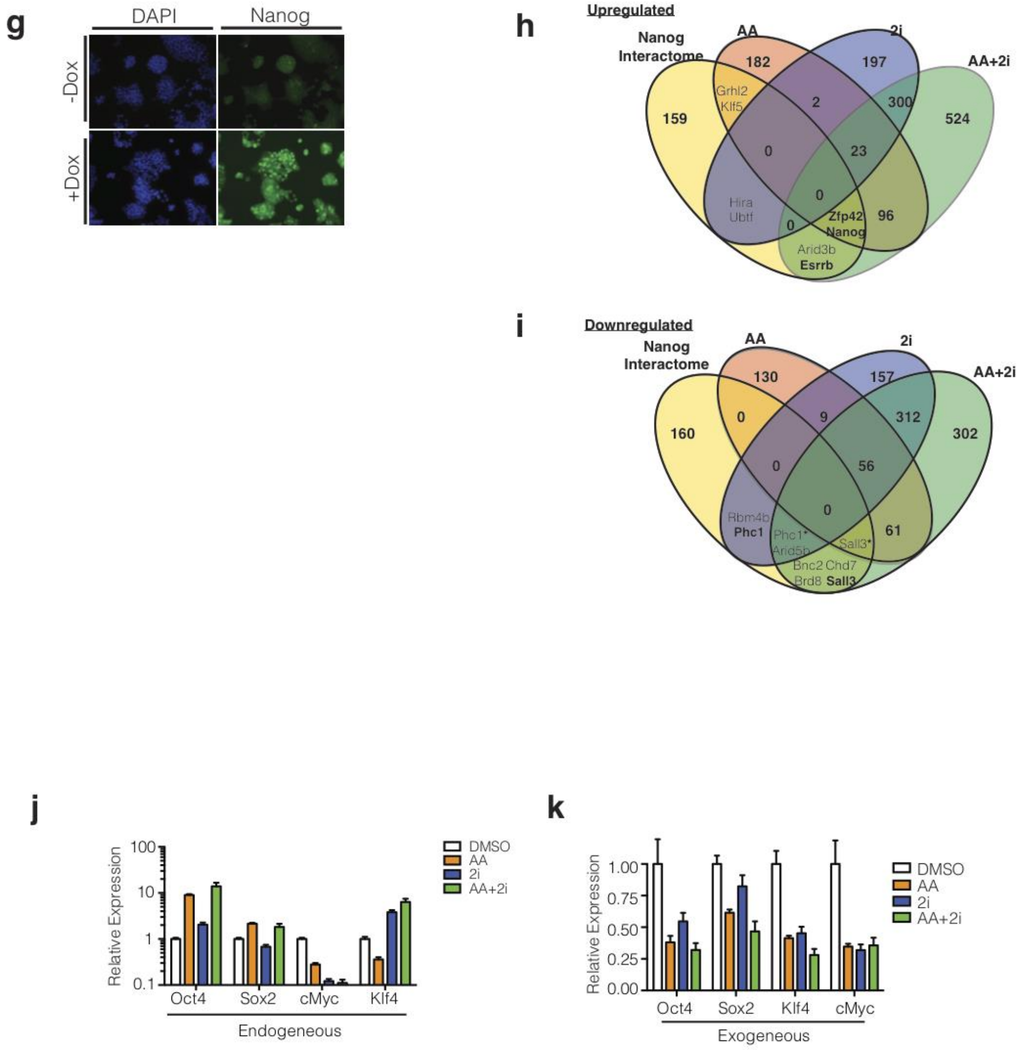

Supplementary Figure 5: Greater connectivity in AA+2i network

a) Network of genes DE and two-fold upregulated in the AA+2i condition generated from overlaying gene expression data onto STRING interaction database. Zoomed out view of snapshot presented in Fig 5a. **b)** Zoomed out view of network of genes DE and two-fold downregulated in the AA+2i condition presented in Fig 6a. **c)** Network of genes DE and two-fold upregulated in the AA condition **d)** Network of genes DE and two-fold downregulated in the AA condition. **e)** Network of genes DE and two-fold upregulated in the 2i condition. **f)** Network of genes DE and two-fold downregulated in the 2i condition. **g)** pre-iPSC line with stably integrated doxycycline-inducible Nanog was induced for 48 hours. Panels show Nanog

immunofluorescence only in the presence of dox. **h)** Venn diagram showing overlap of genes that are upregulated in each condition as compared to control, with the Nanog interactome. Nanog interactome was assembled from references [1-4]. Bold genes are > or = 2 fold upregulated. Asterisk represents genes that switch overlap depending on 2-fold cutoff. **i)** As in (H) but for downregulated genes. **j)** qRT-PCR data of endogenous of the reprogramming factors after 2 days of exposure to DMSO, AA, 2i or AA+2i. Error bars represent standard deviation of three replicates. **k)** qRT-PCR data of exogenous expression of the reprogramming factors after 2 days of exposure to DMSO, AA, 2i or AA+2i. Error bars represent standard deviation of three replicates.

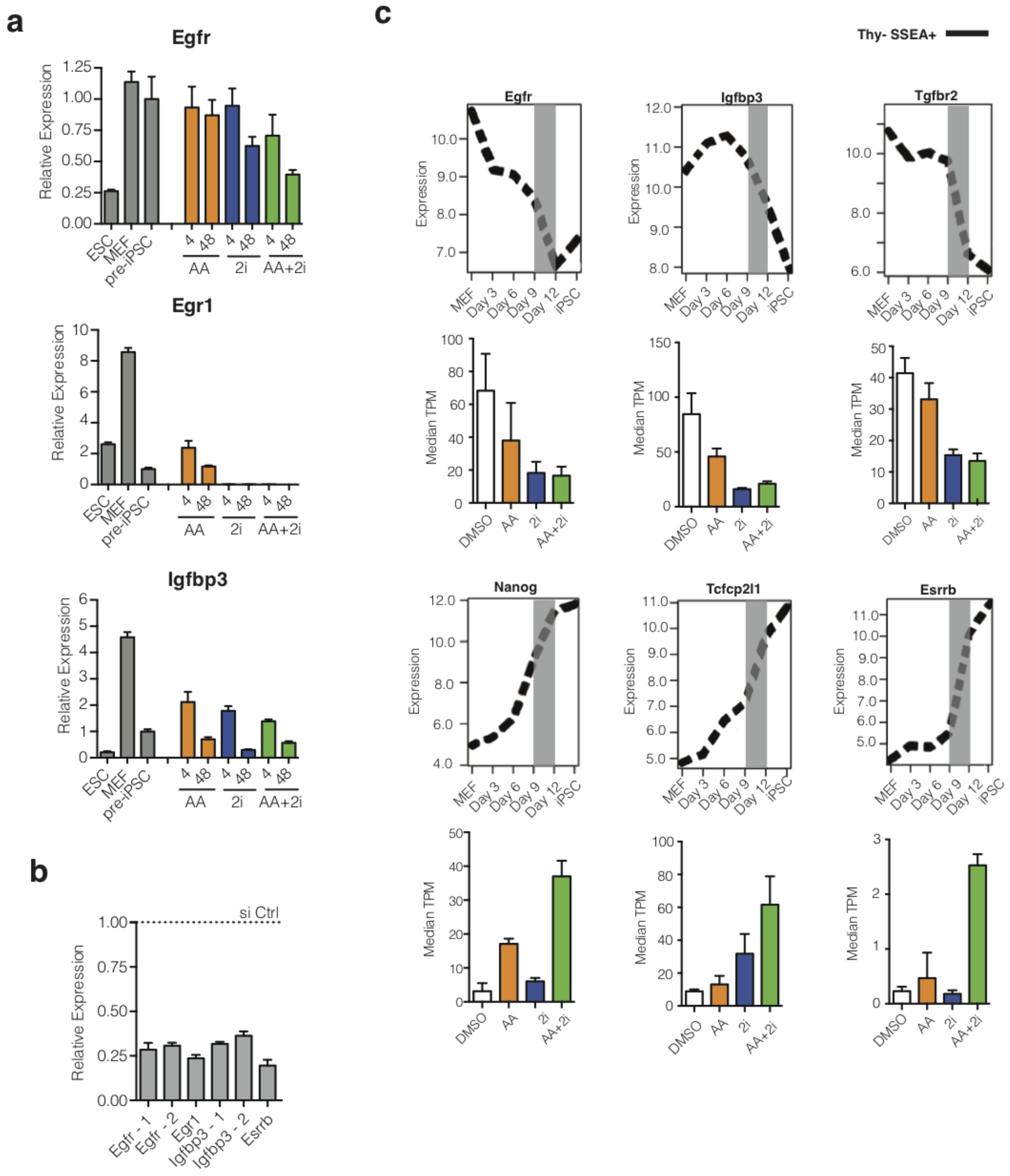

Supplementary Figure 6: pre-iPSC and MEFs undergo similar node transitions.

a) qRT-PCR data at 4 or 48 hours of key downregulated nodes following exposure to AA (orange), 2i (blue) or AA+2i (green). Error bars represent standard deviation of two technical replicates **b)** qRT-PCR data of genes following siRNA treatment. Samples 1 and 2 for Igfbp3 and Egfr represent two different targeting siRNAs used in biological replicate experiments

presented in Fig 6c/6d. Expression for each gene is shown relative to a control siRNA targeting luciferase and represented by the dashed line. Error bars represent standard deviation of two technical replicates. **c)** Key network genes identified were compared to expression changes observed during MEF reprogramming (Polo et al., 2012)[5]. Figures are adapted from Polo et al. Top panels – Expression levels of Thy1-SSEA1+ isolated populations during 12 days of reprogramming. Pre-iPSCs are Thy1- SSEA1+ and closely resemble cells undergoing day 9 to day 12 transition represented by the gray box. Bottom panels – Expression values after 2-day exposure to DMSO, AA, 2i or AA+2i. Values are the median transcripts per million (TPM) across 3 replicates from RNA-sequencing data. Note that data for Egr1 and Igf1r was not present in the Polo et al. microarray dataset.

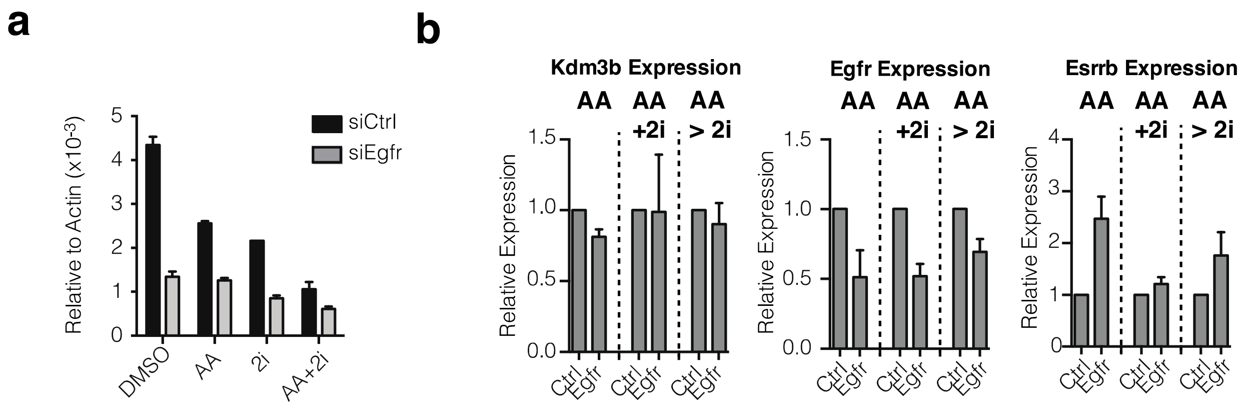

Supplementary Figure 7: a) Expression of Egfr at 48 hours in the indicated conditions in mock transfected or Egfr siRNA transfected in comparison to actin, which was set to 1. Note that the levels of Egfr are depleted in all conditions as compared to DMSO alone. **b)** Egfr expression, for a second distinct siRNA as compared to the one in Fig 7D, after siRNA transfections targeting control (anti-luciferase), Kdm3b, Egfr and Esrrb were performed on days -1, and +1 of exposure to AA alone (day 2 levels) or AA+2i (day 2 levels) or exposure to AA for 2 days followed by switch to 2i for 2 days (day 4 levels) . Error bars represent standard deviation of four replicates.

Supplementary Reference

1) Polo, J.M., Anderssen, E., Walsh, R.M., Schwarz, B.A., Nefzger, C.M., Lim, S.M., Borkent, M., Apostolou, E., Alaei, S., Cloutier, J., et al. (2012). A Molecular Roadmap of Reprogramming Somatic Cells into iPS Cells. Cell *151*, 1617–1632.
